# Realizing the potential of social determinants data in EHR systems: A scoping review of approaches for screening, linkage, extraction, analysis, and interventions

**DOI:** 10.1017/cts.2024.571

**Published:** 2024-10-10

**Authors:** Chenyu Li, Danielle L. Mowery, Xiaomeng Ma, Rui Yang, Ugurcan Vurgun, Sy Hwang, Hayoung K. Donnelly, Harsh Bandhey, Yalini Senathirajah, Shyam Visweswaran, Eugene M. Sadhu, Zohaib Akhtar, Emily Getzen, Philip J. Freda, Qi Long, Michael J. Becich

**Affiliations:** 1 Department of Biomedical Informatics, University of Pittsburgh School of Medicine, Pittsburgh, PA, USA; 2 Institute for Biomedical Informatics, University of Pennsylvania, Philadelphia, PA, USA; 3 Department of Biostatistics, Epidemiology and Informatics, University of Pennsylvania, Philadelphia, PA, USA; 4 Centre for Quantitative Medicine, Duke-NUS Medical School, Singapore, Singapore; 5 Department of Psychiatry, University of Pennsylvania, Philadelphia, PA, USA; 6 Department of Computational Biomedicine, Cedars-Sinai Medical Center, Los Angeles, CA, USA; 7 Institute of Health Policy Management and Evaluations, University of Toronto, Toronto, ON, Canada; 8 Kellogg School of Management, Northwestern University, Evanston, IL, USA

**Keywords:** Social determinants of health, electronic health records, health equity, natural language processing, social risk factors

## Abstract

**Background:**

Social determinants of health (SDoH), such as socioeconomics and neighborhoods, strongly influence health outcomes. However, the current state of standardized SDoH data in electronic health records (EHRs) is lacking, a significant barrier to research and care quality.

**Methods:**

We conducted a PubMed search using “SDOH” and “EHR” Medical Subject Headings terms, analyzing included articles across five domains: 1) SDoH screening and assessment approaches, 2) SDoH data collection and documentation, 3) Use of natural language processing (NLP) for extracting SDoH, 4) SDoH data and health outcomes, and 5) SDoH-driven interventions.

**Results:**

Of 685 articles identified, 324 underwent full review. Key findings include implementation of tailored screening instruments, census and claims data linkage for contextual SDoH profiles, NLP systems extracting SDoH from notes, associations between SDoH and healthcare utilization and chronic disease control, and integrated care management programs. However, variability across data sources, tools, and outcomes underscores the need for standardization.

**Discussion:**

Despite progress in identifying patient social needs, further development of standards, predictive models, and coordinated interventions is critical for SDoH-EHR integration. Additional database searches could strengthen this scoping review. Ultimately, widespread capture, analysis, and translation of multidimensional SDoH data into clinical care is essential for promoting health equity.

## Introduction

The concept of social determinants of health (SDoH) recognizes that health is shaped not only by biological factors or access to medical care but also by the social, economic, and physical conditions that shape people’s lives [1]. Research in disciplines such as public health, sociology, economics, and medicine shows that the circumstances in which people live have a significant impact on shaping patterns of health and well-being [2]. The World Health Organization (WHO) defines SDoH as “*the conditions in which people are born, grow, live, work, and age, along with the wider set of forces and systems shaping the conditions of daily life”* [3] (WHO SDoH concepts see Table 1). These determinants are broadly categorized into five interdependent domains that form the structural and social hierarchies in society: economic stability, neighborhood and built environment, health care access, education access and quality, and social and community context [4,5].

**Table 1.** World Health Organization (WHO)–Social Determinants of Health (SDoH) data components. EHR = electronic health record.

SDoH concept	WHO–SDoH domains	Common EHR representations
Economic Stability	Income, employment, financial resources, and basic needs	Employment status field; Insurance type; ZIP code (proxy for area-level socioeconomic status); Financial resource strain screening questions; Housing instability screening
Neighborhood and Built Environment	Neighborhood (zip code), transportation, food access, environmental conditions	Patient address/ZIP code; Transportation needs screening; Food insecurity screening; Environmental exposure history
Health and Health Care	Insurance status (type, coverage, payer, provider), behavioral and mental health	Insurance information fields; Primary care provider; Mental health screening results; Substance use screening; Medication adherence data
Education	Attainment, level, language	Education level field; Primary language field; Need for interpreter services; Health literacy screening
Social and Community Context	Race/ethnicity, connections, marital status	Race/ethnicity fields; Marital status; Social support screening questions; Domestic violence screening; Sexual orientation and gender identity fields

Specifically, adverse SDoH like poverty, unequal access to education, lack of public resources in neighborhoods, high crime rates, racial segregation, and pollution are all strongly associated with higher rates of morbidity, mortality, and health risk behaviors across populations [1]. On the other hand, protective and promoting SDoH like higher household income, safe green spaces, strong social support, affordable nutrition options, and accessible transportation, have been linked to positive health indicators ranging from self-rated health status to lower diabetes and longer life expectancy [6].

Health outcomes are greatly influenced by more than just clinical encounters; indeed, research suggests that only about 20% of a person’s health outcomes can be attributed to clinical care [7,8], the majority of health outcomes are determined by a combination of individual behaviors and various external factors that are collectively referred to as SDoH. These “causes of the causes” of health are estimated to account for up to 55% of population health variation in high-income countries, though some estimates suggest they may account for as much as 70–80% [8]. Aspects of physical environment, socioeconomic status, race, and gender contribute to systemic inequities that manifest as adverse outcomes. This makes social determinants fundamental considerations for achieving health equity and improving overall population health [1].

### SDoH-driven translational research: deriving and translating health data to actionable knowledge into clinical care

**Figure 1.**
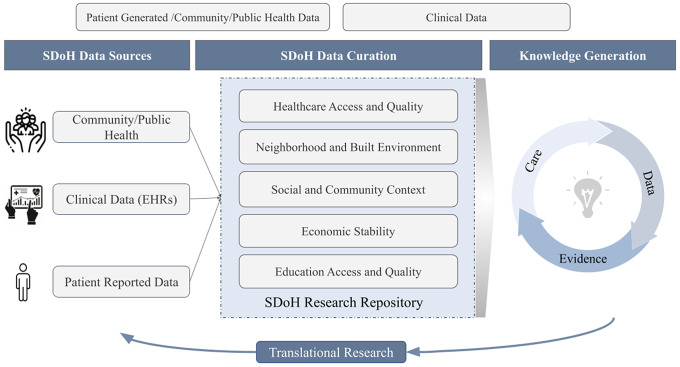
Data-to-knowledge-to-action workflow for translating social determinants of health (SDoH) into clinical care. EHR = electronic health record.

Incorporating SDoH into clinical practice is essential for health equity, but these determinants are rarely consistently recorded in electronic health records (EHRs). Researchers have implemented various approaches to standardize SDoH data collection, study the generated data, and apply the knowledge to improve care (see Fig. 1). SDoH data, collected from surveys [9], EHR modules [10], and patient-reported outcomes [11], can be aggregated into a unified repository for targeted research. However, data collection, integration, and utility remain inconsistent across systems. To be useful, data must be integrated and standardized using ontologies [12], common representations [13], and value sets [14]. Integrated SDoH data can be studied with health outcomes and linked to programs [15]. Leveraging SDoH data in EHRs can activate embedded tools like alerts and flags, such as guiding interventions like nutrition assistance based on hunger scores [16], referring patients to community health workers for those in disadvantaged neighborhoods [17], and creating high-risk patient panels for targeted care [18]. This integration facilitates personalized care management and health equity through patient-centric technologies [19,20], fostering a learning health system.

### Challenges and barriers

Integrating SDoH data into EHRs is crucial for promoting health equity, but significant barriers exist [3,21]. Key challenges include incomplete and inconsistent data in structured fields [21,22], varying screening tools and data standards limiting interoperability [23], and difficulties in consistently gathering and updating SDoH data due to clinical and administrative workflows [7].

Confidentiality rules and patient mistrust regarding information sharing also hinder SDoH data sharing [24]. Privacy-preserving tools like DeGAUSS [25] offer a promising approach by enabling secure and privacy-preserving sharing of SDoH data through geographical aggregation and statistical noise. However, the adoption of such tools may face challenges related to organizational policies, data governance, and stakeholder trust. Addressing these issues requires a multi-pronged approach involving policy change, system redesign, and community engagement [26].

Studies have shown that SDoH factors are commonly discussed in clinical encounters but rarely documented in structured fields, consistent with gaps in systematic SDoH data capture in EHRs [27]. While natural language processing (NLP) approaches can help extract SDoH data from free-text notes, a more robust data collection and integration framework is needed. Connecting patients experiencing SDoH to relevant programs and services is critical, but determining patient eligibility and accessibility can be challenging. Clinical decision support systems and digital health technologies can assist healthcare professionals in making appropriate recommendations [28].

Our scoping review addresses the lack of a comprehensive understanding of SDoH-EHR integration by providing an integrated framework spanning data capture, analytics, and applications. We aim to identify best practices, gaps, and future directions by addressing key questions related to standardized tools, external data linkage, NLP methods, and the impact of harmonized SDoH data on health outcomes and interventions.

## Method

### Scoping literature review

We conducted a scoping review to explore the current landscape of SDoH data integration into EHRs. Scoping reviews are particularly useful for examining emerging evidence when the specific questions that can be addressed by a more precise systematic review are not yet clear [29–31]. The five predetermined focus areas aligned with the SDoH research pipeline and key steps in SDoH-EHR integration, spanning from data capture to analytics and applications. These areas guided the analysis of included studies, and our approach is consistent with the methodological framework for scoping reviews.

### Search strategy

The literature search was conducted in PubMed, a widely recognized database for biomedical literature, on 2023 May 8th. We utilized Medical Subject Headings (MeSH) terms to refine our search, focusing on articles indexed with terms “Electronic Health Records” and “Social Determinants of Health.” This combination was chosen to specifically target studies that discuss the intersection of EHRs with SDoH (Search strategy see Supplement Table 1 in Supplementary Material 1).

### Screening and selection process

**Figure 2.**
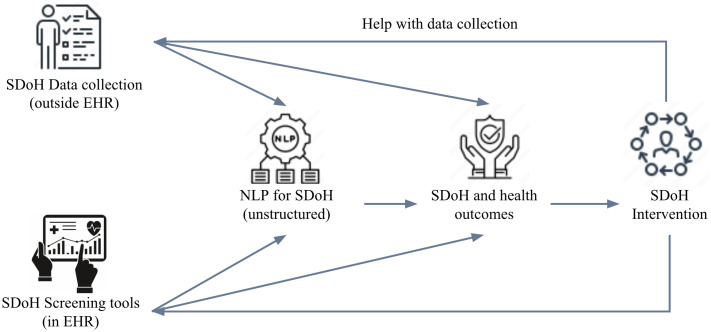
Five social determinants of health categories describing the data workflow from data capture efforts to interventions. EHR = electronic health records; NLP = natural language processing.

The screening process involved three phases. In phase one, papers were categorized into five non-mutually exclusive topics (depicted in Fig. 2):

- **SDoH Screening Tools and Assessments**: Papers discussing various tools and methodologies for screening SDoH.
- **SDoH Data Collection and Documentation**: Studies focusing on how SDoH data are collected and documented within EHR systems.
- **Use of NLP for SDoH:** Research exploring the application of NLP techniques to identify and extract SDoH information from unstructured EHR data.
- **Associations between SDoH and Health Outcomes:** Papers examining the relationship between SDoH and various health outcomes.
- **SDoH Interventions:** Studies that evaluate the effectiveness of interventions aimed at addressing SDoH within healthcare settings.

In phase two, aligned with PRISMA guidelines [32], the screening process involved an initial title/abstract review phase led by author C.L. (criteria see Supplement Table 2 in Supplementary Material 1) to categorize papers into one or more of the 5 topics. Targeted metadata extraction was performed by assigned reviewers as follows: Screening Assessments (R.Y.), SDoH Data Collection (C.L.), NLP Approaches (C.L.), and Interventions (X.M.). The SDoH and Outcomes papers were randomly assigned to the broader reviewer pool (C.L., R.Y., S.H., D.L.M., U.V., H.K.D.) for metadata extraction. Additional irrelevant studies were excluded in this second phase. The full-text metadata extraction phase allowed confirmation of accurate categorization and extraction, with discrepancies resolved through consensus meetings. Evidence synthesis leads included: C.L. for SDoH Screening tools and SDoH and health outcomes, D.L.M for SDoH Data collection, NLP for SDoH, X.M. for SDoH Interventions, overseen by senior authors M.J.M. and D.L.M.

In phase three, senior authors conducted evidence synthesis and conflict resolution, validating phase one and two results. The PRISMA flow diagram [32] was used to depict the screening process.

*The multi-stage process with independent categorization, full-text metadata extraction, and consensus meetings embedded quality checks aligning with scoping review best practices.*

## Results

In this section, we present the findings of SDoH in the EHR according to five domains of interest.

### Data collection and synthesis

**Figure 3.**
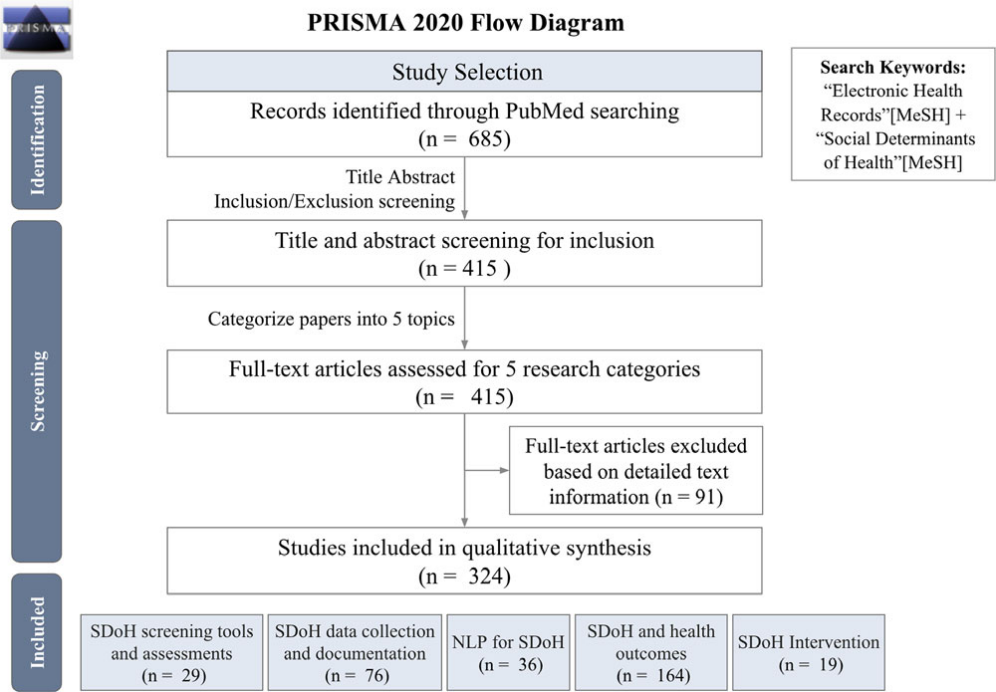
PRISMA 2020 flow diagram. EHR = electronic health records; NLP = natural language processing.

We identified a total of 685 articles through the PubMed query. After reviewing the titles and abstracts screening, 415 articles were included. Of these 415 articles, 324 articles included full text for qualitative synthesis. The reviewed articles were then classified according to SDoH in the EHR domains. The majority of articles focused on SDoH and health outcomes, SDoH data collection and documentation followed by NLP for SDoH, and SDoH screening tools and assessments. In the following sections, we reviewed the major themes and highlighted works for each of the five SDoH in the EHR domains (see Fig. 3, percentage see Supplement Table 3 in Supplementary Material 1).

### SDoH screening tools

We included 29 papers (details see Supplement Material 2 – Meta Data) incorporating SDoH Screening tools into EHRs in our review. The majority of the studies utilized homegrown tools for screening SDoH, reflecting the need for tailored approaches and the limitations of existing standardized tools in certain contexts Some studies [27,33–35] developed their own questionnaires and screening sets, reflecting a trend toward customized tools tailored to specific healthcare settings or populations. Vendor-specific tools, like the two-item screening tool [36] integrated into Epic SDoH Wheel, were less common but still present. The screened determinants varied, but common factors included housing, food insecurity, transportation, and mental health indicators like stress and depression.

Studies targeted a diverse range of populations. For example, children were the focus in some studies [33,37], while adults were the primary subjects in other studies [34,38]. Various healthcare settings were represented, from primary care clinics [39,40] to emergency departments [41], as well as school-based clinics [35]. This diversity indicates the widespread recognition of SDoH’s importance across different medical environments, underscoring its growing relevance throughout the healthcare spectrum.

Active screening methods, where healthcare providers proactively administered questionnaires or interviews, were predominant (*n* = 28) [27,34]. Passive methods like the analysis of EHR data [42] were less common. However, the utilization of EHR data for passive screening indicates a potential to streamline the process in the future. While many studies focused on personal health determinants (*n* = 19), others also assessed structural determinants like housing quality and social networks [33,40,43]. A limited number of studies (*n* = 8) investigated both personal and structural determinants.

**Figure 4.**
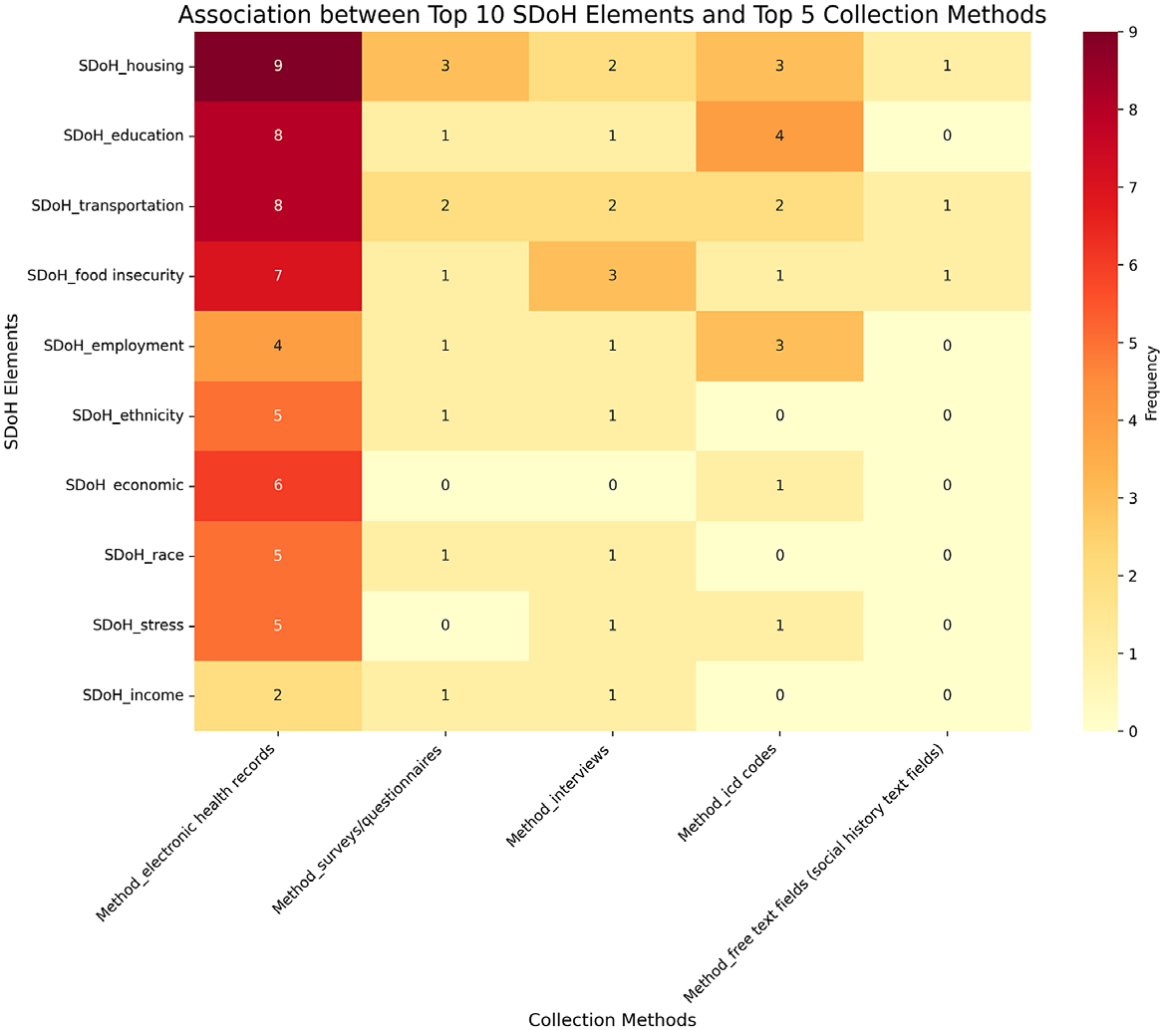
Heatmap showing the frequency of association between the top 10 social determinants of health (SDoH) elements and the top five data collection methods.

The heatmap (Fig. 4) shows EHRs as the dominant data source, with surveys, interviews, and ICD codes used for specific elements. Free text fields were underutilized, suggesting an opportunity for leveraging unstructured data. Housing was studied through the most diverse methods. The heatmap highlights the importance of EHRs and the need for diverse, integrated approaches.

### Challenges and opportunities

The reviewed studies collectively highlight the challenges in standardizing SDoH screening across various contexts but also point toward the potential benefits of such screenings to patient care. The diversity in approaches reflects the complexity of addressing SDoH in clinical practice. However, it also demonstrates a concerted effort toward more comprehensive patient care. The prevalence of homegrown tools [33,35,40,44], indicates a trend toward customization, tailored to specific patient populations and healthcare settings. This is likely due to the unique needs and circumstances of different patient demographics. The variability in tools and approaches (e.g., the number of questions in studies [27,45], and the use of paper-based vs. EHR-based tools [34,39] highlight the challenges in standardizing SDoH screening. This variability could impact the comparability of data and the scalability of successful approaches. Despite the challenges, the focus on SDoH screening illustrates a shift toward more personalized patient care. Recognizing and addressing social and behavioral factors [35,46] can lead to more effective healthcare interventions and better health outcomes.

### SDoH data collection and documentation

In our review, we identified 76 articles (see Supplement Material 2 – Meta Data) describing SDoH data collection and documentation practices. These studies focused on engagement with populations and leveraged a variety of technologies to support collection and documentation processes.

The reviewed studies demonstrated that various technologies were employed to support the collection and documentation of SDoH data. Screening tools and questionnaires were also commonly used, often integrated directly into the EHR system. For example, Boston Medical Center’s THRIVE tool [47] utilized a paper screening form that was entered into the EHR by medical assistants, while the University of California, San Diego [48] used a local EHR database (Epic) to collect SDoH data through ICD codes and discrete data fields. The OCHIN network of community health centers [49] developed an EHR-based screening questionnaire to assess various SDoH domains, and Wake Forest School of Medicine [50] employed a tablet-based digital health system to screen for food security, housing, and transportation needs.

In addition to EHRs and screening tools, some studies leveraged web-based platforms and applications for SDoH data collection. For instance, the COMPASS-CP study[51] used a web-based application and iPad application to capture patient-reported outcomes related to physical, mental, and social well-being, as well as financial challenges and caregiver needs.

NLP techniques were also employed to extract SDoH information from unstructured EHR data. The OSF HealthCare System[52] utilized the Pieces NLP system to identify SDoH factors from EHR notes, demonstrating the potential of NLP in automating the extraction of SDoH data from free-text clinical documentation.

Furthermore, studies used geocoding techniques to link patient addresses with external data sources, such as census tract socioeconomic data and community-level characteristics. The Envirome Web Service [53], developed by Children’s Mercy Hospital, integrated census tract data with patient EHRs using real-time geocoding, enabling a more comprehensive understanding of patients’ social and environmental contexts.

Our review identified diverse methods for SDoH data collection and integration. Nine studies incorporated qualitative approaches, including interviews with patients and clinicians, community engagement initiatives, focus groups, and town hall meetings [54–62]. These methods provided rich, contextual information about SDoH factors and their impacts on health outcomes.

Technology-assisted collection methods were also prevalent, with seven studies utilizing various tools for SDoH data gathering. These included paper-based entry, iPads/tablets, patient or clinician-facing web portals, and other web-based toolkits and forms [50,51,63–67].

Several studies (*n* = 6) made use of publicly available, external data resources to infer structural SDoH information for a given population. The most common external SDoH data sources linked to EHRs were US census and community survey data (at both patient/individual and area/neighborhood levels), administrative data/claims records, and disease registries. Commonly linked community surveys and systems include the Behavioral Risk Factor Surveillance System, the National Health and Nutrition Examination Survey, the National Health Interview Survey [68], the National Institutes of Health PROMIS® (Patient-Reported Outcomes Measurement Information System) [69], the National Survey of Children’s Health [70], the Center for Disease Control Youth Risk Behavior Surveillance System [71], the Center for Medicare and Medicaid Services (CMS) Accountable Health Communities’ Health-Related Social Needs Screening Tool [72], the National Center for Education Statistics, the Uniform Crime Reports, and the American Community Survey [73–76]. Longitudinal study data included the National Longitudinal Study of Adolescent to Adult Health (Add Health) [77]. Other administrative data sources included the Healthcare Cost & Utilization Project (HCUP) Nationwide Readmissions Database [78], claims data [79], and Medicaid data warehouse [80]. Few studies describe use of disease-specific registries e.g. cancer registries such as SEER-CMS, SEER-Medicare, and SEER-Medicaid [81]. While these sources may not directly capture SDoH information, they can provide proxy measures related to healthcare utilization patterns, access to care, and socioeconomic status. However, the use of administrative and claims data for SDoH analysis has limitations, as they may lack the granularity and specificity of data collected directly from patients or through dedicated SDoH screening tools.

Eight studies describe methods for inferring structural SDoH using geocoding of patient addresses and linking to public census tract data [53,66,82,83] These studies employed various geocoding techniques to convert patient addresses into geographic coordinates, which could then be mapped to specific census tracts or other geographic units. For example, the Envirome Web Service, developed by Children’s Mercy Hospital, used real-time geocoding to link patient addresses with census tract data [53]. This geocoding approach enabled the integration of information related to neighborhood and community-level characteristics (e.g., SES, crime incidence, and health facility locations) [74,85,86] and neighborhood factors (e.g., poverty level, education, employment status, etc.) [47,48,53,68,87–89]. By linking patient locations to area-level SDoH data, these studies were able to provide a more comprehensive understanding of patients’ social and environmental contexts, even when individual-level SDoH data were not available in the EHR.

External data provided various socioeconomic factors (income, education, employment, poverty level, air quality), neighborhood variables (segregation, safety, and walkability), and health behaviors (diet, exercise, and smoking) [47–49,52,54,79,90–97]. These complemented and expanded the individual-level SDoH data (food/housing security, transportation, interpersonal violence, etc.) captured directly in EHRs [98–100]. A small subset of studies (*n* = 3) aimed to integrate EHR, genomic, and public health data to examine the intersection of lifestyle, genetics, and environmental influences [48,101,102].

### Challenges and opportunities

Although these works highlight the potential for study of personal and structural SDoH, there is considerable effort for systematically collecting, linking, and analyzing SDoH data from external sources together with EHR data at the community, state, and national levels [103,104]. The adoption of common data models to improve standardization and interoperability of collected SDoH data remains limited [105]. Moreover, there is a scarcity of research demonstrating how this integrated information could be leveraged to connect individuals with identified SDoH risk factors to appropriate social programs.

### NLP in SDoH

In our review, we identified 36 articles (details see Supplement Material 2 – Meta Data). describing NLP methods for powering SDoH studies. Many SDoH elements are captured in clinical free-text notes, such as progress notes, discharge summaries, and social work assessments. These unstructured data often contain rich, contextual information about patients’ social circumstances that may not be fully captured in structured fields. For example, free text might include detailed descriptions of a patient’s living situation, family dynamics, or barriers to accessing care. In contrast, structured data elements typically consist of predefined fields or checkboxes in the EHR, such as standardized screening questionnaires or ICD-10 Z-codes for social factors [106–108]. The use of NLP techniques is crucial for extracting and analyzing SDoH information from these unstructured sources, as it allows researchers to access a wealth of data that might otherwise remain untapped. NLP can identify mentions of social factors, assess their relevance, and even determine the severity or impact of these factors on the patient’s health.

**Figure 5.**
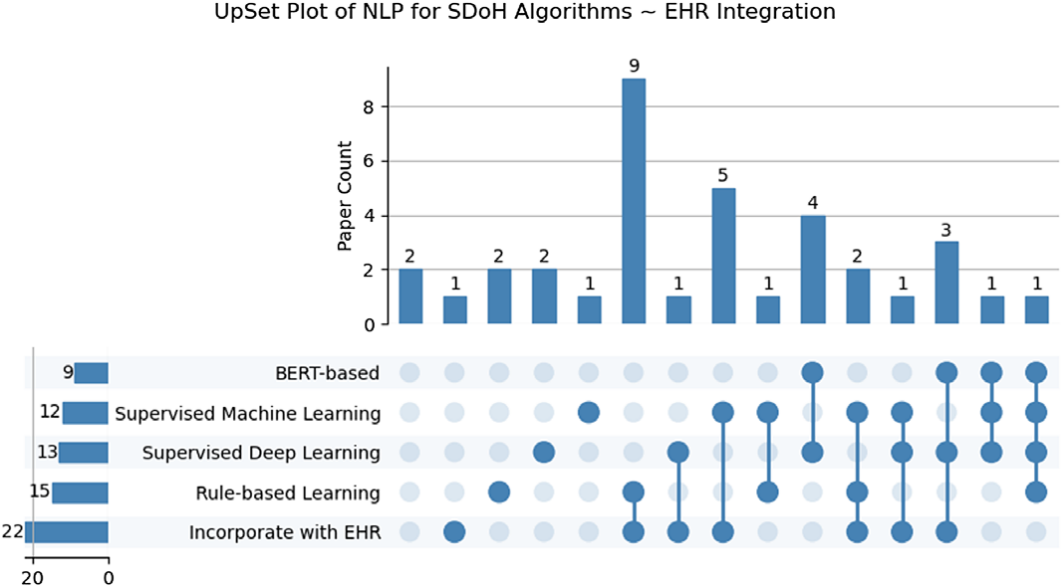
UpSet Plot of natural language processing for social determinants of health (SDoH) algorithms ∼electronic health record (EHR) integration. *Outlining the distribution of papers in which each approach/method individually and in combination was described in the study. *Supervised machine learning includes traditional machine learning methods (naive bayes, support vector machine, logistic regression, random forest, etc), excluding neural networks and pretrained approaches.

Several studies focused on lexicon development using methods such as lexical associations, word embeddings, term similarity, and query expansion. Lexicons and regular expressions have been demonstrated to extract SDoH and psychosocial risk factors [18,109–112], learn distinct social risk factors by mapping them to standard vocabularies and code sets including ICD-9/10, ICD Z codes, **U**nified **M**edical **L**anguage **S**ystem, and SNOMED-CT. Most articles (*n* = 15) describe rule-based approaches using regular expressions and/or hybrid machine learning methods leveraging platforms. Five articles highlighted well-known rule-based toolkits and platforms adapted with lexicons and regular expressions for SDoH extraction including Moonstone, **Easy C**linical **I**nformation **E**xtraction **S**ystem, **M**edical **T**ext **E**xtraction, **R**easoning and **M**apping **S**ystem, **Q**ueriable **P**atient **I**nference **D**ossier, and **Cl**inical **Eve**nt **R**ecognizer [18,109–112]. Other articles (*n* = 7) describe rule-based systems paired with traditional machine learning approaches i.e., an ensemble, particularly using NLP systems such as **G**eneral **A**rchitecture for **T**ext **E**ngineering, **C**linical **L**anguage **A**nnotation, **M**odeling, and **P**rocessing Toolkit, **E**xtr**a**ct **S**DOH from **E**HRs, Yale **c**linical **T**ext **A**nalysis and **K**nowledge **E**xtraction **S**ystem, **Re**lative **Hou**sing **S**tability in **E**lectronic **D**ocumentation, and toolkits such as spaCy and medspaCy in conjunction with conditional random fields and support vector machines (SVM) [113–116]. In contrast, several investigators have leveraged open-source NLP toolkits like spaCy and medspaCy without supervised learners to extract SDoH variables [117–119]. Other studies (*n* = 19) have solely leveraged traditional supervised and unsupervised learning techniques, SVM, logistic regression (LR), Naïve Bayes, Adaboost, Random Forest, XGBoost, Bio-ClinicalBERT, Latent Dirichlet Allocation, and bidirectional long short-term memory [125] to extract and standardize social and behavioral determinants of health (SBDoH), e.g., alcohol abuse, drug use, sexual orientation, homelessness, substance use, sexual history, HIV status, drug use, housing status, transportation needs, housing insecurity, food insecurity, financial insecurity, employment/income insecurity, insurance insecurity, and poor social support. In more recent years, nine studies have focused on the training and tuning of deep learning approaches, primarily transformer-based [126–135] approaches i.e., Bidirectional Encoder Representations from Transformers (BERT), RoBERTa, BioClinical-BERT models for extracting SBDoHs including relationship status, social status, family history, employment status, race/ethnicity, gender, social history, sexual orientation, diet, alcohol, smoking housing insecurity, unemployment, social isolation, and illicit drug use—from clinical notes, PubMed, among other specialized texts, e.g., LitCOVID [126–135]. The frequency of papers for SDoH extraction NLP algorithms within EHR systems, highlighting the combinations and intersections of utilized methodologies can be found in Fig. 5.

### Challenges and opportunities

Although these works highlight the potential for extracting SDoH from texts, several challenges remain. Few studies focused on lexicon development, make use of standard terminologies for encoding SDoH data, and explore deep extraction and representation of SDoH attributes and relationships. Also, many studies focus on extraction and encoding SDoH data from a single site and fail to assess the portability of methods to new textual data sources beyond clinical notes and PubMed articles such as digital technologies and chatbots. The introduction of shared datasets like the Social History Annotated Corpus is an important step toward demonstrating generalizability of NLP-powered, SDoH extraction systems. Emerging generative models may also improve upon the state-of-the-art demonstrated by common shared task datasets.

### SDoH and health outcomes

In our review, we identified 164 articles (details see Supplement Material 2 – Meta Data). describing SDoH and health outcomes. SDoH and health outcome studies examined a wide variety of health-related events and outcomes in relation to SDoH factors. A predominant focus was on infectious disease outcomes, with 16 studies examining drivers of COVID-19 hospitalization, mortality, treatment disparities, and differences in positivity rates across social groups [140–149]. Another major category included healthcare utilization metrics like preventable hospital readmissions (*n* = 11) [140–149], ED reliance ( *n* = 16) [173], and telehealth adoption [174–184]. Beyond infectious outcomes and healthcare utilization, studies also assessed chronic disease control across conditions like diabetes (*n* = 11) [174–184], hypertension [187,188], kidney disease [150,179,189–193], and obesity (*n* = 7) [171,194–203], along with risk factors like elevated blood pressure and cardiovascular events. Some studies focused on cancer (*n* = 11) screening, diagnoses, treatment disparities, and survival outcomes [150–155], while others addressed mental health (*n* = 6) indicators [204] ranging from dementia incidence [205,206] to suicide (*n* = 2) risk factors [207,208]. Additional outcomes evaluated included maternal morbidity (*n* = 2) [177,209] and pediatric health metrics, ranging from vaccine completion rates to epilepsy-related consequences.

The most common quality measures reported were standardized condition control thresholds like HbA1c levels for diabetes control [195], blood pressure (*n* = 5) levels for hypertension control, and established cancer staging guidelines [205]. Some studies used validated risk prediction models for outcomes like hospital readmissions (*n* = 5), suicide risk [210,211], or mortality (*n* = 6). Beyond clinical indicators, several studies incorporated validated SDoH indexes like the Area Deprivation Index [212], Social Deprivation Index [213], and CDC’s Social Vulnerability Index [163,183,184,196,203,214–221]. In terms of analysis approaches, common methods included multivariate regression models like LR (*n* = 13) [142,200,216] and Cox proportional hazards models (*n* = 3) [219] to assess adjusted outcome associations with SDoH factors. Other advanced techniques included machine learning algorithms [222], geospatial analysis for clustering [154], and phenome-wide association studies [223,224].

### SDoH interventions

A total of 19 papers (details see Supplement Material 2 – Meta Data) collected supplementary SDoH data to support population health intervention initiatives targeting hospitals/clinics (*n* = 10) or communities (including primary care, *n* = 9) at the meso (institution) level. Two articles discussed policy potential and proposed policy reform at the macro (system) level [225]. The majority of the selected research (*n* = 16) focused on implementing a social and healthcare-supportive program to address the social needs of the target population. Interventions were implemented in various settings for hospital-based initiatives, including posthospital discharge [226], the emergency department [227–231], and clinics specializing in different medical disciplines [232–235]. On the other hand, community-based initiatives concentrated mainly on integrating interventions into primary care services [236,237].

The social and healthcare supportive programs included a range of initiatives designed toward improving community health. These initiatives encompassed the introduction of new healthcare programs [229,236,238], health education and coaching [232], the strengthening of medical-legal partnerships [233,235,239], the enhancement of integrated care planning [226,240,241] and the improvement of patient navigation [227,236,238]. Only a few papers (*n* = 3) have examined the potential of incorporating the family or social support element into their intervention design [225]. Meanwhile, four studies investigated the potential for enhancing resource allocation through surveying outcomes. The improvement objectives encompassed the allocation of staff and equipment [228], the enhancement of patient navigation [230,242], and the transformation of health service practices [237].

Our review identified a range of target populations receiving SDoH interventions. Several interventions focused on specific demographic groups, including racial/ethnic minorities (e.g., Blacks for hypertension control) [227,228], specific age groups (both pediatric and adult populations) [231], and women’s health [225]. Health condition-specific interventions were also common, targeting chronic diseases such as heart failure [237], COPD [233], diabetes [230], and hypertension [231,235], as well as mental health conditions [239] and specific diseases like lupus [234,239,241,242]. Many studies focused on vulnerable or underserved populations.

Health outcomes, such as improvements in health metrics, reductions in disease incidence, changes in vital signs, and quality of life, are commonly used as measures to determine the feasibility of initiating an intervention (*n* = 10). Several studies have also assessed social SDoH in relation to patient satisfaction and acceptability [243–245]. Since most programs were new efforts, it was not possible to determine the effectiveness of the intervention in the short term, nor could the potential generalizability be assessed.

## Discussion

This scoping review set out to map SDoH-EHR integration literature across five key domains: structured data capture tools, external data linkage approaches, NLP-based extraction techniques, and applications for outcomes analysis, and health care interventions. Our results synthesized major themes and collective gaps within each sphere. Regarding our first aim, predominant tailored screening instruments enable assessment but standardization barriers persist. For the second objective, enriching patient profiles via claims and census linkage shows promise but systematic consolidation is lacking. On research question three, rule-based systems boast precision while neural networks improve unstructured element recognition – yet reproducibility hurdles remain. Finally, concerning predicting outcomes and targeting programs, consistent risk evidence conflicts with implementation uncertainty. Across the five domains, our review highlights the progress made in SDoH data integration, while also identifying critical gaps and challenges. We provide a comprehensive assessment of the current state of SDoH research, from data collection and analysis to the development and evaluation of interventions. Our findings underscore the need for standardized approaches, improved data interoperability, and more rigorous evaluation of SDoH interventions.

By synthesizing insights from diverse research areas, we offer a roadmap for advancing SDoH-EHR integration. This cross-domain perspective reveals the interdependencies between different aspects of SDoH research and practice, emphasizing the importance of a holistic, “full-stack” approach. Our review lays the foundation for future work in this field, guiding researchers and practitioners in their efforts to leverage SDoH data for promoting health equity and improving patient outcomes.

### Key findings by theme

#### SDoH screening tools

The studies revealed variety of screening tools to assess patients’ SDoH across diverse healthcare settings. The most prevalent SDoH domains screened included housing instability, food insecurity, transportation and utility service needs, interpersonal safety, financial strain, social isolation, health literacy, and education level. Notably, the majority utilized homegrown instruments rather than standardized tools, with PRAPARE, National Academy of Medicine recommendations, and CMS Accountable Health Communities screening tool being the most commonly referenced standardized options.

These tools were tested across various settings, from primary care clinics to emergency departments and inpatient units. While most relied on active screening during visits, some explored passive methods like paper questionnaires or electronic tablets. The instruments primarily focused on individual-level SDoH, with a minority attempting to capture community or structural factors.

This widespread implementation reflects a growing recognition of upstream factors in shaping health outcomes. By systematically documenting social and environmental impacts on health, providers and researchers aim to address root causes of health disparities, aligning with population health management and preventive medicine principles.

Researchers found SDoH screening feasible and effective in identifying unmet social needs across diverse populations and implementation strategies. However, further research is warranted to develop optimal referral systems and interventions for identified needs, evaluate the impact of SDoH screening on patient outcomes, or develop evidence-based interventions that effectively address identified social needs. This will help to fully realize the potential of this upstream approach in improving overall health outcomes and reducing health disparities.

#### SDoH data collection and documentation

Our review reveals a rich landscape of SDoH data collection and integration efforts, as summarized in Table 2. The integration of external data sources with EHRs has significantly enhanced the capture of SDoH, providing critical information on socioeconomic position, neighborhood characteristics, and health behaviors [246]. This integration enables more holistic patient profiling, supporting risk stratification, outcomes studies, and health equity initiatives.

**Table 2.** Social Determinants of Health (SDoH)-driven translational research: deriving and translating actionable knowledge into clinical care. NLP = natural language processing.

SDoH Screening Tools	SDoH Data Collection and Documentation	NLP in SDoH	SDoH and Health outcomes	SDoH Interventions
**SDoH Domains Assessed** Most common elements screened were housing instability/insecurity, food insecurity, transportation needs, utility needs, financial resource strain, interpersonal safety issues. Other factors included social isolation, health literacy, education level, employment status. Tools targeted a range of personal and structural determinants. **Screening Approaches** Majority utilized home-grown, customized questionnaires rather than standardized validated instruments. Most common existing tools referenced were PRAPARE, CMS Accountable Health Communities, and NAM recommendations. Both active screening by staff and passive self-report methods used. **Settings** Implemented across varied clinical settings - primary care, EDs, inpatient units, community health centers. Some population-based screening at schools or by telephone. **Effectiveness** Broad feasibility shown across populations and settings to identify unmet social needs. More evidence needed regarding interventions to address identified needs. **Key Next Steps** Expanding regular screening with validated tools tied to follow-up resources. Increasing structural screening and community-clinical linkages. Integrating social needs data with EHRs and longitudinal outcomes.	**Data Sources** The most common external SDoH data sources linked to EHRs were census and community survey data (at both patient/individual and area/neighborhood levels), administrative data like claims records, and disease registries. Other sources included geospatial data, crime statistics, built environment data, education data, and proprietary population health databases. Some studies used qualitative interviews or surveys to gather additional patient SDoH information not found in the EHR. **SDoH Elements** External data provided various socioeconomic factors (income, education, employment, poverty level), neighborhood variables (segregation, safety, walkability), and health behaviors (diet, exercise, smoking). These complemented and expanded the individual-level SDoH data (food/housing security, transportation, interpersonal violence, etc.) captured directly in EHRs. **Linkage Approaches** Technical approaches to integrate external SDoH data with EHRs included geocoding patient addresses, aggregating community variables to patients, and direct linkage using unique identifiers. Integration enabled richer patient- and population-level SDoH data for risk stratification, outcomes research, social care coordination, and addressing health disparities. **Gaps & Challenges** More work is still needed to systematically collect, link, analyze, and act upon SDoH data from external sources together with EHR data.	**Methods Used** Most common NLP approaches were rule-based systems using regular expressions or lexicons and supervised machine learning models like CNNs, LSTMs, SVMs, and ensembles. Recent studies utilized pretrained contextual models like BERT which showed promising performance. Both generic NLP libraries (spaCy) and custom systems tailored to SDoH were tested. **Performance** Accuracy ranged widely based on model type and SDoH category but precision and recall generally over 80% for housing, occupation, and some social risks. Simple models had high precision but lower sensitivity in identifying key SDoH entities. Advanced neural networks improved recall. Overall, NLP could identify more SDoH data than structured EHR fields alone. **Applications** Inferring patients’ social risks, socioeconomic status, and exposures to guide interventions. Predicting outcomes like hospital readmissions, suicide risk, and future healthcare utilization. Enriching datasets for disparities research and population health surveillance. **Limitations & Next Steps** Better standardized corpora for model development and testing are needed. Methods to efficiently integrate NLP pipelines into clinical workflows rather than one-off analyses. Domain ontologies and shareable custom systems for SDoH extraction from notes.	**Outcomes Assessed** Most common outcomes examined were healthcare utilization (ED visits, hospitalizations, readmissions), chronic disease control (diabetes, hypertension, CVD), and COVID-19 severity. Other outcomes included cancer screening/treatment, obesity/BMI, mortality, mental health, substance use disorders, and patient-reported metrics. **SDoH Factors** Frequently measured SDoH elements were neighborhood disadvantage, food/housing insecurity, access to care/insurance, education, income, and social support. Race/ethnicity, immigrant status, and geographic factors were also analyzed as social determinants. **Analytical Approaches** Regression models evaluated associations between SDoH factors and outcomes. Some prediction models incorporated both clinical and social variables. Studies linked area-level SDoH data from census and surveys to individual-level EHR data. **Key Findings** Multiple studies found socioeconomic deprivation, insecure housing, lack of social support, and similar factors increased risk for adverse outcomes. But overall evidence was mixed, highlighting context-specific impacts. More research is needed on mechanisms.	**Types of Interventions** Most common interventions were social programs like community health initiatives, resource referrals/patient navigation services, integrated care management, and group education sessions. Some studies allocated additional resources like medical staff or equipment. A few tested policy changes or system-level practice transformations. **Implementation Levels** Interventions operated at the community, hospital/clinic, or health system level. Community programs enabled broader reach and incorporation of public health principles. Clinic-based initiatives allowed better integration with healthcare delivery. **Components** Roughly half emphasized family/peer support and social connections as part of the intervention. **Outcomes** Knowledge, self-efficacy, and resource utilization were commonly measured process outcomes. Clinical outcomes like chronic disease control, medication adherence, and health behaviors were assessed in some studies. Cost savings and healthcare utilization were less frequently examined. **Effectiveness** Most interventions showed some benefits but had limited generalizability due to small samples or single health systems. Overall evidence was mixed and highlighted implementation barriers regarding sustainability, adoption, and cost.

The SDoH data collection efforts span a wide range of domains, from housing and food insecurity to education and employment status. The diversity of data sources – including census data, community surveys, and administrative claims – reflects the multifaceted nature of social determinants. However, this diversity also underscores the challenges in standardizing data collection and integration practices.

Several initiatives have been developed to address these challenges through the creation of common data elements (CDEs) and standardized models [247], including the Gravity Project [248], the PhenX Toolkit [249], All of Us [250], and USCDI [250], and the extensions to the OMOP Common Data Model [49,251,252]. These efforts aim to enable consistent data collection, facilitating better understanding of SDoH contributions to health inequities and improving data sharing. However, a gap persists between available standardized elements and their implementation in practice, contributing to heterogeneity in SDoH data collection and documentation. Limited adoption of CDEs can be attributed to technical challenges in integrating new data structures into existing EHR systems, resource constraints, staff training needs, and the diverse nature of SDoH factors across populations and healthcare contexts.

Technical approaches for integrating external SDoH data with EHRs have employed geocoding of addresses, aggregation of community measures, and linkage based on unique identifiers. While progress has been made, further research must promote systematic collection, analysis, and application of integrated data sources. Key steps include implementing reliable linkage mechanisms for disparate datasets and embedding multidimensional patient social profiles within clinical decision tools and workflows [253–255].

Only through purposeful integration and translation efforts can external SDoH data fully support identification of at-risk populations, patient-centered risk assessments, and targeted community-clinical interventions.

#### NLP in SDoH

A range of NLP approaches have been leveraged to identify critical social determinants from unstructured clinical notes. These methods can be broadly categorized into: 1. Rule-based systems using expert-curated lexicons and regular expressions; 2. Supervised machine learning models (e.g., convolutional neural networks, recurrent neural networks); 3. Advanced contextual embedding models (e.g., BERT).

Both generic NLP software libraries and custom systems tailored to social and behavioral health domains have been implemented. While reported accuracy metrics vary by model type and target social determinants, precision and recall generally exceed 80% for key factors like housing insecurity and occupations. Simpler models often demonstrate high precision, while recent neural networks improve sensitivity in capturing key entities from free-text fields.

Importantly, these NLP approaches recognize more patient social factors than structured EHR data alone, enabling richer risk assessments and interventions. However, challenges remain in standardization and integration into clinical workflows.

In the future, we can focus on developing better-standardized corpora for reusable NLP systems in social domains, integrating validated SDoH screening workflows into routine practice, improving ontologies and shareable custom systems, enhancing linkages to longitudinal outcomes, and conducting rigorous assessments of multi-sector SDoH interventions and their specific mechanisms of impact.

These efforts will facilitate more comprehensive and effective identification and addressing of SDoH across diverse populations.

#### SDoH and health outcomes

This review provides insights into current approaches and gaps in research on SDoH and health outcomes. Most studies were retrospective analyses examining links between social determinants and health issues, including neighborhood disadvantage, food and housing insecurity, healthcare access barriers, healthcare utilization, chronic illness control, and infectious diseases. A smaller number of studies assessed mental health, cancer, and mortality. This distribution of research focus highlights areas where more investigation is needed to provide a comprehensive understanding of SDoH impacts across all health domains.

COVID-19 has significantly impacted SDoH research, stimulating greater attention to health disparities. Studies consistently found higher COVID-19 risks and deaths among minorities, low-income groups, and those with prior conditions. Researchers leveraged diverse data sources, including medical records, census indices, and surveys, to quantify the disproportionate pandemic burden on disadvantaged groups. Some studies displayed sophisticated applications of predictive analytics and machine learning to model disease dynamics. This crisis has expanded SDoH data infrastructure and methodology while underscoring long-term disparities. Assessing pandemic response and recovery across social levels is critical, as disruptions may exacerbate existing health inequities among vulnerable groups.

Methodologically, regression modeling was commonly used to characterize adjusted outcome associations. However, more advanced analytics and predictive modeling were less prevalent. This gap presents an opportunity for more sophisticated computational research to uncover precise interactions between SDoH and health outcomes.

Moving forward, key areas for development include standardizing processes for SDoH data collection and integration into medical records, shifting from predominantly observational analyses to more interventional studies, and translating research findings into community initiatives for at-risk groups. Developing analytic guidelines to navigate the complexities of real-world SDoH data and creating standardized frameworks for SDoH data analysis in healthcare are also crucial.

These advancements are essential for health outcomes research, providing a foundation for more effective, evidence-based interventions and policies that consider the broad influences of social factors on health. By addressing these challenges, we can better leverage SDoH data to inform healthcare decisions and strategies, ultimately working toward reducing health disparities and improving population health.

#### SDoH interventions

Interventions addressing health care-related issues occur at micro (patient care), meso (healthcare institutions), and macro (healthcare policy) levels [256,257]. Our review found that SDoH recognition primarily facilitates interventions at the meso level, including primary care and specialist referrals, patient navigation services, integrated care management, group education sessions, and resource allocation. Fewer studies reported macro-level policy changes or micro-level interventions emphasizing family/peer support and social connections for individual patients.

While many interventions showed some benefit, their generalizability was often limited due to narrow focus within single health systems (citation needed). This limitation is particularly relevant in the US, where health delivery systems are often fragmented into regional networks (citation needed). Implementing interventions for vulnerable populations presents unique challenges, as demographics vary across communities depending on cultural and geographical factors [258,259].

Healthcare professionals must recognize that identifying SDoH within a community is only the initial stage. Establishing connections between individuals facing both health and social issues can be challenging due to various barriers. Building trust with vulnerable individuals is an ongoing process requiring sustained social and material support from healthcare professionals and community social workers. Creating effective regional support networks necessitates lasting partnerships with organizations possessing resources to address SDoH-related challenges, such as housing and transportation [24].

To enhance intervention effectiveness, mature plans with SDoH collection tools embedded in EHR systems should be adopted and tailored to the target population’s needs. Careful selection of platforms for survey distribution and data storage is crucial to prevent duplication of effort, ensure data integrity, and promote program sustainability. It’s imperative to collect intervention-informing data directly from the affected population. Incorporating patient feedback is essential for achieving optimal results. Pilot surveys can be used to pretest data collection instruments, allowing for refinement based on patient input. This collaborative approach helps create a more supportive environment for vulnerable individuals and mitigates unforeseen obstacles [261].

Along with these methods, many interventions have been tried to help with known social problems and imbalances. Some of these are community health programs, help finding resources, patient navigation services, unified care management models, and educational meetings with peer support. Vulnerable groups have been given extra resources like more medical staff or tools in some studies. System-level policy changes have also been implemented to promote health equity [260].

Future research should focus on rigorous evaluation of health outcomes improvement to ensure the long-term success and widespread applicability of SDoH interventions [261,262].

### Cross-cutting insights

Despite progress in SDoH research and implementation, several challenges persist across domains. These challenges highlight the need for an integrated approach to advance the field.

Standardization remains a critical issue in SDoH integration. The current variability in screening tools, data collection methods, and documentation practices [263] hinders comparability and generalizability of findings. There’s an urgent need for standardized corpora, data elements, and workflows across all aspects of SDoH integration to facilitate more robust and comparable research.

Data integration presents another significant challenge. Presently, expertise and data often reside in silos, with screening, linkage, extraction, analysis, and intervention programs operating independently [264]. Breaking down these silos to create a comprehensive platform spanning from data collection to application is crucial for optimizing SDoH efforts. As research continues, embracing interoperable design principles and controlled evaluation around representative datasets, model transparency, and equitable outcomes remains vital [265].

Technological advancements offer both opportunities and challenges. While NLP and machine learning show promise in SDoH identification and analysis, their application remains limited. Few studies have employed advanced predictive modeling techniques, highlighting an area for growth. The recent exponential development in large language models (LLMs) presents new opportunities for SDoH entity recognition across contexts [266,267].

Longitudinal studies are crucial for understanding the long-term impact of SDoH interventions. Enhanced linkages to longitudinal outcomes are needed to fully grasp the effects of interventions over time. This requires rethinking workflows to integrate contextual data into real-world utilities [49].

As the field evolves, embracing interoperable design principles, controlled evaluation around representative datasets, and a focus on equitable outcomes will be vital. The integration of advanced technologies like LLMs must be balanced with ethical considerations and rigorous validation to ensure their reliable and equitable application in SDoH contexts.

By addressing these cross-cutting issues, the field can move toward more comprehensive, effective, and equitable integration of SDoH in healthcare, ultimately improving population health outcomes and reducing health disparities.

### Limitations

This scoping review faces certain limitations in comprehensively capturing the state of SDoH data integration into EHRs. Relying solely on PubMed for literature searches and limiting the results to English papers may introduce selection bias, omitting potentially relevant research indexed in other databases. Supplementing with sources like SCOPUS or Web of Science may have revealed additional insights and applications. Additionally, our search strategy relies on the MeSH terms “SDoH” and “EHR” in our search strategy. While using standardized subject headings helps retrieve relevant articles indexed in MEDLINE and PubMed, it may have limited the scope of our search. Future research could expand the search strategy to include specific SDoH factors and compare the results with our current findings. This approach may provide a more comprehensive understanding of the literature on SDoH and EHR integration, particularly for studies conducted before the widespread use of the term ‘SDoH’.

Due to resource constraints, the metadata extraction from the final set of included studies was completed by a single reviewer. Having dual independent extraction with consensus meetings is ideal to ensure accuracy and completeness of scoping review data abstractions. The feasibility and impact of implementing a second reader should be evaluated in future updates to strengthen robustness.

Our review methodology, which relies on published literature, may not fully capture the landscape of SDoH screening tools used in clinical practice. Many healthcare institutions have implemented SDoH screening within their EHR systems without publishing these efforts in academic literature. This gap between published research and actual clinical practice means our review may underestimate the prevalence and variety of SDoH screening tools in use. Our findings primarily reflect tools that have been reported in academic literature, which may disproportionately represent novel or custom-developed instruments rather than more commonly used, commercially available tools.

Finally, heterogeneity across settings, populations, tools, and outcomes creates complexity in evaluating SDoH-EHR integration maturity. Varying implementation stages and study designs introduce difficulty in benchmarking best practices. The scoping methodology prioritized inclusiveness over appraising integration quality, leaving gaps in assessing real-world effectiveness. Capturing nuanced, multidimensional integration processes by diverse healthcare systems persists as a challenge, though framework refinement helps structure insights.

Future research could benefit from alternative methodologies to capture a more comprehensive picture of SDoH screening practices and data integration. This might include surveys of healthcare institutions, analysis of EHR vendor data, or case studies of health systems’ unpublished screening practices. Such approaches could help bridge the gap between published literature and real-world implementation of SDoH screening tools and data integration practices.

## Conclusion

Overall, while collecting patient social contexts shows immense potential to rectify health disparities, realizing these possibilities requires ongoing informatics innovation alongside economic investments and policy reforms targeting root societal drivers. This review contributes an evidence base for such continued progress in wisely applying multidimensional SDoH data to promote health equity.

The integration of SDoH data into healthcare practice holds transformative potential for addressing health disparities. Realizing this potential demands continued innovation, strategic investment, and policy evolution, guided by the evidence and insights garnered from comprehensive SDoH research.

## Supporting information

Li et al. supplementary material 1 Li et al. supplementary material

Li et al. supplementary material 2 Li et al. supplementary material

## Supplementary material

The supplementary material for this article can be found at https://doi.org/10.1017/cts.2024.571
