# Supplementary material for: Realizing the potential of social determinants data in EHR systems: A scoping review of approaches for screening, linkage, extraction, analysis, and interventions
Source: J Clin Transl Sci. 2024 Oct 10;8(1):e147. doi: 10.1017/cts.2024.571 (PMC11523026; doi:10.1017/cts.2024.571)

Supplement

**Supplement Table 1** - Search MEDLINE Strategy (2023. May 8th on PubMed )

| (#1) AND (#2) |
| --- |
| (health information exchange [tw] OR hie [tw] OR rhio [tw] OR regional health information organization [tw] OR hl7 [tw] OR health level seven [tw] OR unified medical language system [majr] OR umls [tw] OR loinc [tw] OR rxnorm [tw] OR snomed [tw] OR icd9 cm [ti] OR icd 9 cm [ti] OR icd10 [ti] OR icd 10 [ti] OR metathesaurus [tw] OR patient card [tw] OR patient cards [tw] OR health card [tw] OR health cards [tw] OR electronic health data [tw] OR personal health data [tw] OR personal health record [tw] OR personal health records [tw] OR Health Records, Personal [Majr] OR Health Record, Personal [Majr] OR ehealth [tw] OR e-health [tw] OR medical informatics application [mh] OR medical informatics applications [mh] OR medical records system, computerized [mh] OR medical records systems, computerized [mh] OR computerized patient medical records [tw] OR automated medical record system [tw] OR automated medical record systems [tw] OR automated medical records system [tw] OR automated medical records systems [tw] OR computerized medical record [tw] OR computerized medical records [tw] OR computerized patient records [tw] OR computerized patient record [tw] OR computerized patient medical record [tw] OR electronic health record [tw] OR electronic health records [tw] OR Electronic Health Record [Majr] OR Electronic Health Records [Majr] OR electronic patient record [tw] OR electronic patient records [tw] OR electronic medical record [tw] OR electronic medical records [tw] OR electronic healthcare records [tw] OR electronic healthcare record [tw] OR electronic health care record [tw] OR electronic health care records [tw] OR archives [majr] OR ehr [tw] OR ehrs [tw] OR phr [tw] OR phrs [tw] OR emr [tw] OR emrs [tw] OR Health Information Systems [Majr] OR health information interoperability[mh] OR health information interoperability[tw]) AND (medical record [ti] OR medical records [mh] OR medical records [ti] OR patient record [ti] OR patient records [ti] OR patient health record [ti] OR patient health records [ti] OR patient identification system [mh] OR patient identification systems [mh] OR Patient Outcome Assessment[Majr] OR Patient Discharge Summaries[Majr] OR healthcare record [ti] OR healthcare records [ti] OR health care record [ti] OR health care records [ti] OR health record [ti] OR health records [ti] OR hospital information system [tw] OR hospital information systems [tw] OR umae [ti] OR attitude to computers [mh] OR medical informatics [ti] OR Information Technology[mh] OR Information Technology[tw])) OR ((medical records systems, computerized [majr] OR medical records systems, computerized [mh] OR computerized patient medical record [tw] OR computerized patient medical records[tw]OR automated medical record system [tw] OR automated medical record systems [tw] OR automated medical records system [tw] OR automated medical records systems [tw] OR computerized medical record [tw] OR computerized medical records [tw] OR computerized patient records [tw] OR computerized patient record [tw] OR patient generated health data[mh] OR patient generated health data[tw] OR electronic health record [tw] OR electronic health records [tw] OR electronic patient record [tw] OR electronic patient records [tw] OR electronic medical record [tw] OR electronic medical records [tw] OR electronic healthcare records [tw] OR electronic healthcare record [tw] OR electronic health care record [tw] OR electronic health care records [tw] OR unified medical language system [majr] OR unified medical language system [tw] OR umls [tw] OR loinc [tw] OR rxnorm [tw] OR snomed [tw] OR icd9 cm [ti] OR icd 9 cm [ti] OR icd10 [ti] OR icd 10 [ti] OR metathesaurus [tw] OR ehr [tw] OR ehrs [tw] OR phr [tw] OR phrs [tw] OR emr [tw] OR emrs [tw] OR meaningful use [tiab] OR meaningful use [tw] OR Meaningful Use [Majr]) |
| Social Determinants of Health [MeSH] |

**Supplement Table 1.2 – Inclusion Exclusion criteria**

| Inclusion Criteria: |
| --- |
| Original empirical research studies, including case studies, technical reports, and database studies  Studies centrally focused on integrating/analyzing SDoH data elements in electronic health records systems  Addressed one or more of following defined five domains  Provided details on methods, data sources, outcomes, targets related to SDoH factors  Published in the English language |
| Exclusion Criteria: |
| ● Review papers, editorials, opinion pieces, conceptual frameworks  ● Informatic models/approaches without tie to real EHR/health database  ● Purely qualitative studies lacking SDoH-EHR data focus  ● Biomedical studies incorporating socioeconomic variables, but lacking central SDoH-EHR focus  ● Lacking details on techniques/sources for ingesting/analyzing SDoH data  ● Study protocols without substantive findings section |

**Supplement Table 2** - Screening Included MEDLINE Paper by Domain:

| Domain | Title/Abstract screening | | Full-text screening | |
| --- | --- | --- | --- | --- |
|  | Count (sum = 415) | Percentage | (n = 324) | Percentage |
| SDoH and health outcomes | 168 | 40.5% | 164 | 50.6% |
| SDoH data collection and documentation | 132 | 31.8% | 76 | 23.5% |
| NLP for SDoH | 42 | 10.1% | 36 | 11.1% |
| Review Paper | 28 | 6.7% | / | / |
| SDoH screening tools and assessments | 26 | 6.3% | 29 | 9.0% |
| SDoH Intervention | 19 | 4.6% | 19 | 5.9% |

Supplement Table 3 - Meta Data - SDoH Screening and Assessment

Supplement Table 4 - Meta Data - SDoH Data Collection and Documentation

Supplement Table 5 - Meta Data - NLP for SDoH

Supplement Table 6 - Meta Data - SDoH and Health Outcomes

Supplement Table 7 - Meta Data - SDoH Intervention

**Supplement Table 8** - Search EMBASE Strategy (2024. June 8th on EMBASE) and searched NLP for SDoH papers

('electronic health record'/exp OR 'electronic health record'/de) AND 'social determinants of health'/exp AND (2014:py OR 2015:py OR 2016:py OR 2017:py OR 2018:py OR 2019:py OR 2020:py OR 2021:py OR 2022:py OR 2023:py) AND ([embase]/lim OR ([medline]/lim NOT ([embase classic]/lim AND [medline]/lim))) AND ('article'/it OR 'article in press'/it OR 'chapter'/it OR 'conference paper'/it OR 'note'/it)

319 papers were found in EMBASE only, after drop duplications with MEDLINE search, 130 papers were left. After paper title/abstract screening we found 82 papers fit out inclusion/exclusion criteria, among these 82 papers, 9 papers are related to NLP for SDoH section.

EMBASE searched NLP for SDoH papers :

| **ID** | **Title** |
| --- | --- |
| 4 | Natural Language Processing to Identify Social Determinants of Health in Alzheimer's Disease and Related Dementia from Electronic Health Records |
| 26 | Evidence of Housing Instability Identified by Addresses, Clinical Notes, and Diagnostic Codes in a Real-World Population with Substance Use Disorders |
| 27 | Machine Learning Functional Impairment Classification with Electronic Health Record Data |
| 37 | An Explainable Artificial Intelligence Approach for Discovering Social Determinants of Health and Risk Interactions for Stroke in Patients with Atrial Fibrillation |
| 83 | Predictive Analytics in Hiv Surveillance Require New Approaches to Data Ethics, Rights, and Regulation in Public Health |
| 95 | Using Natural Language Processing to Study Homelessness Longitudinally with Electronic Health Record Data Subject to Irregular Observations |
| 136 | Artificial Intelligence and Machine Learning Technologies in Cancer Care: Addressing Disparities, Bias, and Data Diversity |
| 203 | Natural Language Processing and Machine Learning of Electronic Health Records for Prediction of First-Time Suicide Attempts |
| 261 | Towards the Inference of Social and Behavioral Determinants of Sexual Health: Development of a Gold-Standard Corpus with Semi-Supervised Learning |

**Supplement Figure 1** - Included papers by year
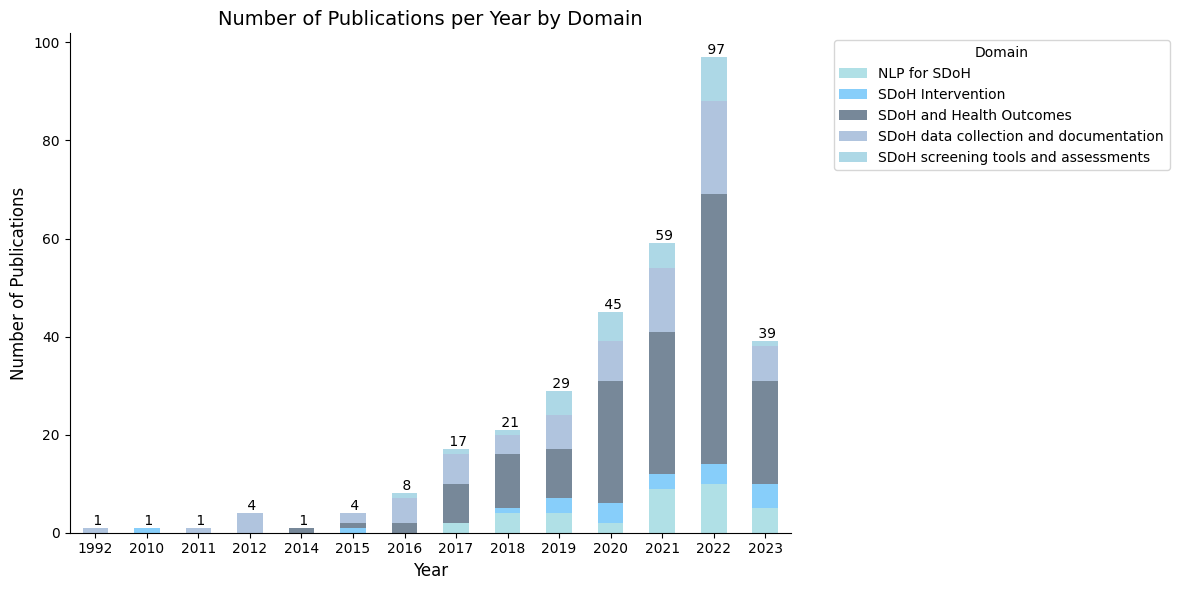


**Supplement Figure 2** - UpSet Plot of SDoH and Health Outoutcomes- Health Events

**
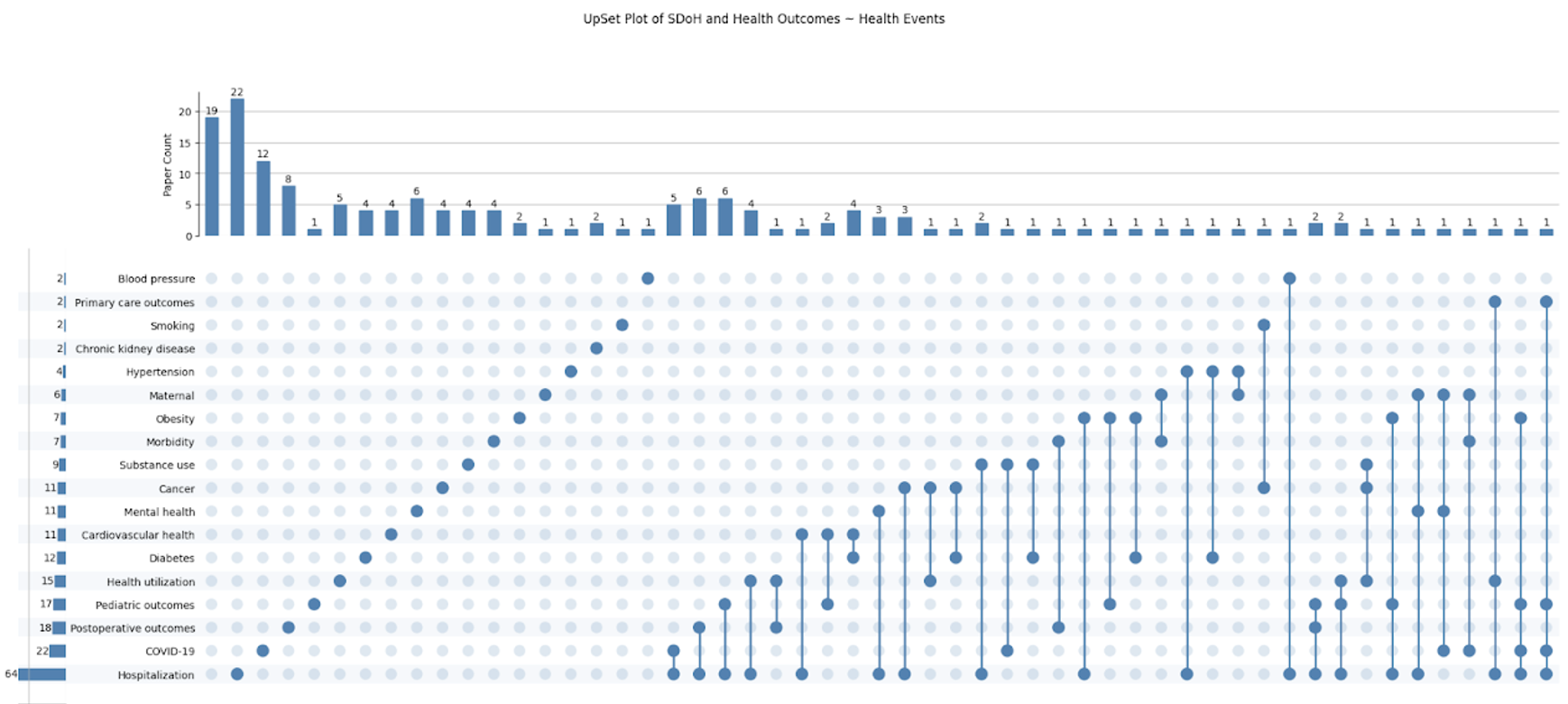
**

**Supplement Figure 3** - Upset Plot of SDoH and Health Outcomes -Study Type


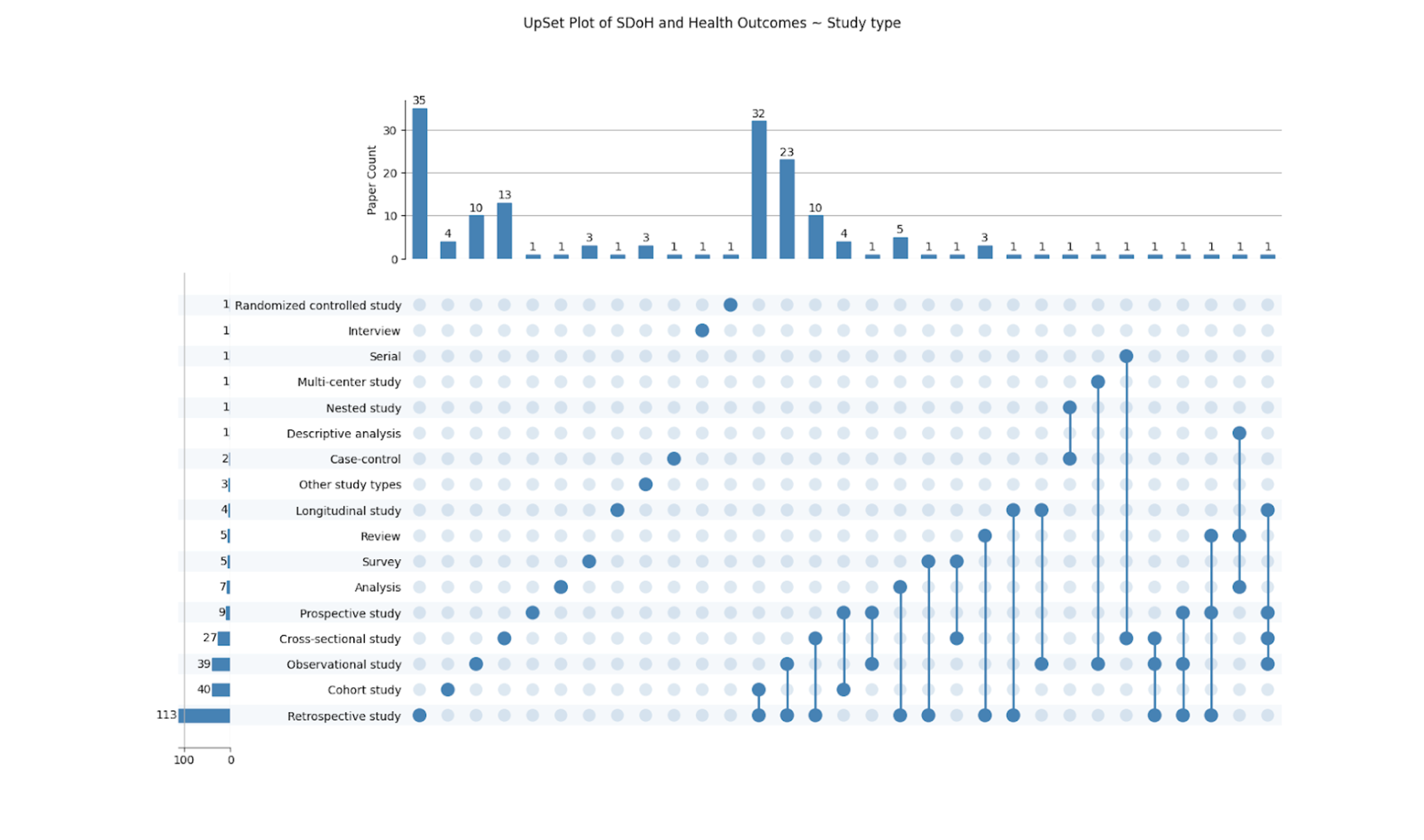

Supplement: Li et al. supplementary material 1 — Li et al. supplementary material [file S2059866124005715sup001.docx]
