# Supplementary material for: Realizing the potential of social determinants data in EHR systems: A scoping review of approaches for screening, linkage, extraction, analysis, and interventions
Source: J Clin Transl Sci. 2024 Oct 10;8(1):e147. doi: 10.1017/cts.2024.571 (PMC11523026; doi:10.1017/cts.2024.571)
Supplement: Li et al. supplementary material 2 — Li et al. supplementary material [file S2059866124005715sup002.pdf]

EndNote_ID	PubMed_ID	Title	Involved Data Elements	Tool Name	Vendor-specific / home-grown	Target population	Screening settings	Active/Passive	personal/structural	personal/structural-clean	Remarks
144	34210842	An Ecological Model to Frame the Delivery of Pediatric Preventive Care	Socioeconomic status, social and physical disorder, access to services and amenities, organization of social networks, safety	Community-level data aggregation	Home-grown	Children	Primary care, community settings, population level	Active	structural	structural	
157	34628675	Evaluation of the Core 5 Social Determinants of Health Screening Tool	Financial resource strain, transportation, stress, depression, intimate partner violence, social isolation, physical activity, alcohol use	11-question paper questionnaire	Home-grown	Adult patients (18+) receiving Medicare wellness, adult wellness, or new patient visits	Single adult ambulatory clinic within a large integrated health network	Active	Personal and structural	both	
209	30201636	Adoption of Social Determinants of Health Ehr Tools by Community Health Centers	Housing, food, transportation, education, income, safety, social connections, stress, depression	14 questions based on PRAPARE and NAM recommendations	Home-grown	Adult patients at community health centers	3 community health centers using shared EHR system	Active - staff administered paper questionnaire	Both personal and structural	both	
217	32350005	Differences in Pediatric Residents' Social Needs Screening Practices across Health Care Settings	Food, housing, education, intimate partner violence, legal needs, maternal depression	Not specified	Not specified	Pediatric patients	Inpatient and outpatient care at an academic children's hospital and affiliated clinics	Active - residents self-reported screening rates	Mainly personal	personal	
219	30898498	Screening for the Social and Behavioral Determinants of Health at a School-Based Clinic	Depression, anxiety, tobacco use, alcohol/drug use, violence, food insecurity, health literacy, nutrition, physical activity, sexual health	Custom screening set assembled from validated tools	Home-grown	Adolescent students	School-based health clinic	Active - administered by provider	Mainly personal	personal	
255	35037885	Social Determinants of Health Screening by Preclinical Medical Students During the Covid-19 Pandemic: Service-Based Learning Case Study	Housing instability, utility strain, food insecurity, transportation, financial resources strain, exposure to violence, childcare, behavioral and mental health	10-item Social Determinants of Health Screening Questionnaire	Home-grown	Patients aged ≥50 years who were due for colorectal cancer screening and did not have insurance	Telephone screening	Active screening	Focused on personal determinants	personal	
259	36779911	Partner-Developed Electronic Health Record Tools to Facilitate Social Risk-Informed Care Planning	Housing insecurity, food insecurity, transportation needs, inability to afford medications/medical care, exposure to violence	Suite of clinical decision support tools embedded in Epic EHR	Home-grown	Patients with hypertension and/or type 2 diabetes in community health centers	Primary care visits	Active screening	Focused on personal determinants	personal	
317	35692849	A Local Perspective into Electronic Health Record Design, Integration, and Implementation of Screening and Referral for Social Determinants of Health	Housing instability, food insecurity, transportation, utilities, interpersonal safety	CMS Health-Related Social Needs (HRSNs) Screening Tool	Home-grown	Medicare and Medicaid beneficiaries	Inpatient, outpatient, emergency department	Active screening	Focused on personal determinants	personal	
328	32663083	Universal Screening of Social Determinants of Health at a Large Us Academic Medical Center, 2018	Housing instability, food insecurity, transportation, utilities, safety	CMS Accountable Health Communities Screening Tool	Home-grown	Dual-beneficiary patients in pediatrics, internal medicine, obstetrics/gynecology primary care practices	Primary care practices	Active screening	Focused on personal determinants	personal	
365	34952582	Evaluation of a Social Determinants of Health Screening Questionnaire and Workflow Pilot within an Adult Ambulatory Clinic	Financial resource needs, transportation needs, stress, depression, intimate partner violence, social connections, physical activity, alcohol consumption	11-question paper questionnaire adapted from Epic EHR system	Home-grown	Adult patients with Medicare wellness, annual, new or transfer patient visits	Ambulatory clinic	Active screening	Focused on personal determinants	personal	
384	35779742	The Utility of the Brokamp Area Deprivation Index as a Prescreen for Social Risk in Primary Care	Food insecurity, utility needs, housing instability, childcare, financial resource strain, transportation challenges, health literacy, social isolation	HLSN	Home-grown	Pediatric Patient Families who spoke English, Spanish, or Arabic and had a child <=12 years receiving primary care	Primary care at a large academic institution	Active	Manily personal	personal	
404	32553071	Implementing a Social Determinants Screening and Referral Infrastructure During Routine Emergency Department Visits, Utah, 2017-2018	Housing and utilities, food assistance, transportation needs, legal resources, mental health and addiction services, medical, dental, and vision insurance, employment services, education and training, and domestic violence and abuse	10 questions based on HealthLeads with modifications	Home-grown	Emergency department patients	Post-Triage, discharge time, check-out	Active	Personal and structural	both	
431	31753281	Variation in Electronic Health Record Documentation of Social Determinants of Health across a National Network of Community Health Centers	Financial resource strain, food insecurity, housing insecurity, Inadequate physical activity, relationship safety, social isolation, stress	15 questions based on PRAPARE and NAM recommendations	Vendor-specific	Primary care or mental or behavioral health visit	EHR-based SDH screening tool in a national network of more than 100 CHCs	Active	Manily personal	personal	
436	36729786	Understanding the Role of a Technology and Emr-Based Social Determinants of Health Screening Tool and Community-Based Resource Connections in Health Care Resource Utilization	Food insecurity, housing instability, utility insecurity, transportation needs, financial instability, violence/abuse, language/educational needs, health literacy, and social connectedness	13-item questionnaire (NowPow)	Not specified	Patients (18 y+) in South Carolina's central (Midlands) and northwestern (Upstate) regions, engaged in one of three care and population management programs	South Carolina's largest private nonprofit health system	Active	Manily personal	personal	
507	30993632	Screening for Housing Instability: Providers' Reflections on Addressing a Social Determinant of Health	Housing	HSCR	Home-grown	Eligible veterans	All outpatient clinics	Active	Personal	personal	
574	28241787	Using Self-Reported Data on the Social Determinants of Health in Primary Care to Identify Cancer Screening Disparities: Opportunities and Challenges	Immigrant status, ethnicity, household income and number of people in the household, sexual orientation, preferred language of communication, and housing status	11-questions surevy	Home-grown	Patients who were eligible for at least one of the three cancer screening types	In the waiting room at routine office visits	Active	Personal	personal	
581	34592935	Implementation of Targeted Screening for Poverty in a Large Primary Care Team in Toronto, Canada: A Feasibility Study	Poverty	2 screening questions	Home-grown	Patients had at least one primary care visit over the three previous years, and were living in the most deprived neighborhoods.	A large family health team	Active	Personal	personal	

EndNote_ID	PubMed_ID	Title	Instution name	Tool Name	SDoH elements included	Collection Method	collection frequency	Interplay with electronic health records.(Yes/No)	linking dataset
128	31095052	Implementing an Ehr-Based Screening and Referral System to Address Social Determinants of Health in Primary Care	Boston Medical Center	THRIVE	Homelessness, housing insecurity, food insecurity, inability to afford medications, lack of transportation, utilities, caregiving, unemployment, education	Paper screening form entered into EHR by medical assistants	One time screening for new patients at first visit	Yes - Screening responses mapped to ICD-10 codes and orders in EHR to print referral guides	
136	34549294	Documentation and Review of Social Determinants of Health Data in the Ehr: Measures and Associated Insights	UCSF Health	Not specified	Housing, food insecurity, transportation, financial strain	Structured fields (patient social history questions, problem list Z-codes), free text fields (social history text, social work notes)	Varied based on data type, some one-time, some per encounter	Yes - focused on documentation in and review of data in the EHR	
141	35446610	Linking Electronic Health Records to the American Community Survey: Feasibility and Process							
149	34312264	Purposeful Incorporation of Patient Narratives in the Medical Record in the Netherlands	Dutch Practice-Based Research Network (FaMe-Net)	Context survey	Education level, employment status, traumatic events/abuse, family medical history	Self-reported survey	Updated every 2 years	Yes - Added to EHR	
156	35662804	Social Determinants of Health Data Availability for Patients with Eye Conditions	University of California, San Diego	Local EHR database (Epic)	Tobacco use, alcohol use, drug use, education, financial strain, living situation, stress level, socialization, physical activity, food insecurity, transportation	ICD codes, discrete data fields	Ongoing during clinical encounters	Yes	
			National Institutes of Health	All of Us Research Program	Demographics, social isolation, overall health, income, housing, healthcare access, food security, education, lifestyle	One-time at enrollment, ongoing	Yes		
165	36454231	Screening for Social Determinants of Health: Active and Passive Information Retrieval Methods	OSF HealthCare System	Pieces NLP system	Social integration, domestic safety, education, financial resources, food insecurity, transportation, housing, stress, behavioral health, substance abuse, disability	Passive - NLP of EHR notes	Passive - Retrospective	Yes	
						Active - Digital screening questionnaire	Active - At clinic visits		
168	29385988	A Patient and Family Data Domain Collection Framework for Identifying Disparities in Pediatrics: Results from the Pediatric Health Equity Collaborative	Multiple pediatric hospitals in US and Canada （PHEC）	Not specified	Race/ethnicity, language, sexual orientation, gender identity, disability, social determinants of health	Survey/questionnaire	Not specified	Yes	

173	35854720	Advancing Interoperability of Patient-Level Social Determinants of Health Data to Support Covid-19 Research	N3C Institutions	THRIVE	Homelessness, food insecurity, trouble paying utilities, trouble paying medications, transportation difficulties, child/elder care challenges, education needs	Paper screening tool, entered into EHR flowsheets	At clinical encounters	Yes
179	32119804	Initiating and Implementing Social Determinants of Health Data Collection in Community Health Centers	8 safety net community health centers in 5 states across the United States	Electronic health record (EHR)-based screening questionnaire developed by OCHIN, Inc. network of community health centers	Financial resource strain, food insecurity, housing insecurity, relationship safety, inadequate physical activity, social connection/isolation, stress	EHR-based screening questionnaire	During clinical encounters	Yes
195	34020026	The Envirome Web Service: Patient Context at the Point of Care	Children's Mercy Hospital, Kansas City, MO	Envirome Web Service (EWS)	Census tract socioeconomic data (income, education, housing, age, race/ethnicity data), food access/food desert data	Real-time geocoding of patient address, linking to publicly available census tract data	In real-time at point of care	Yes - Integrated with Cerner electronic health record via web service
214	33936457	Social Determinant of Health Documentation Trends and Their Association with Emergency Department Admissions	Not specified	ICD-10 Z-codes	Housing, economic circumstances, education, employment, family/social issues, legal issues	ICD-10 Z-codes in administrative claims data	At time of admission	Yes - HCUP Nationwide Readmissions Database used which contains ICD-10 codes from EHRs
216	25217095	Moving Electronic Medical Records Upstream: Incorporating Social Determinants of Health	Case 1: Johns Hopkins Children’s Center<br>Case 2: Utility shut-off protection<br>Case 3: Homelessness, housing instability	Case 1: Social history section in Epic EHR<br>Case 2: As needed<br>Case 3: At clinical encounters	Case 1: Food, transportation, child care, utilities	Case 1: Screening questions in EHR	Case 1: At clinical encounters	Yes
222	36292453	Assessing Alignment of Patient and Clinician Perspectives on Community Health Resources for Chronic Disease Management	University of California, San Francisco	Qualitative interviews, neighborhood tours, clinic observations	Food, physical activity, transportation, community resources, pharmacy access, community cohesion	Semi-structured interviews, neighborhood walking tours, clinic visit observations	One-time	Yes
240	35862115	The Adoption of Social Determinants of Health Documentation in Clinical Settings	Washington University School of Medicine	Not specified	Housing, food, transportation, employment, education, literacy	ICD-10 Z-codes	Not specified	Yes
245	35570965	Examining Social Determinants of Health During a Pandemic: Clinical Application of Z Codes before and During Covid-19	Healthcare facilities in South Carolina	EHR database Z-code	Z55-Z65 ICD-10 CM codes (education, employment, housing, family/social support, etc.)	ICD-10 Z-codes	Not specified	Yes

251	34874813	What Do Ehrs Tell Us About How We Deploy Health Professionals to Address the Social Determinants of Health	University of North Carolina at Chapel Hill	Electronic Medical Record Search Engine (EMERSE)	Food insecurity, housing insecurity	Searched unstructured EHR text notes using keyword bundles related to food and housing insecurity	One-time search	Yes - The study analyzed unstructured EHR text notes
276	36273141	Do Patients Want Clinicians to Ask About Social Needs and Include This Information in Their Medical Record?	University of Pittsburgh	Accountable Care Communities (AHC) Health-Related Social Needs (HRSN) Screening Tool	Housing instability, food insecurity, transportation difficulties, utility assistance needs, interpersonal safety	Web-based survey or telephone interview using AHC HRSN Screening Tool	One-time	No - The social needs data collected was not linked to patients' EHRs
280	36403352	Discuss and Remember: Clinician Strategies for Integrating Social Determinants of Health in Patient Records and Care	Urban teaching hospital in US Midwest	Not applicable	Economic stability, neighborhood and physical environment, education, food, community and social context, healthcare system access	Ethnographic observation, clinician and patient interviews, review of EHR text notes	One-time	Yes - The study analyzed how clinicians document and use SDOH data in EHRs
282	36301647	Leveraging Social Determinants of Health to Reduce Hospital Length of Stay: A Pilot Qi Project for Solid Tumor Oncology Patients During the Covid-19 Pandemic	Medical College of Wisconsin, Froedtert Hospital	Validated SDOH screening survey	Sociodemographic factors, behavioral factors, other risks (see Appendix in article)	Inpatient SDOH survey integrated into electronic medical record	One-time survey completed within 24 hours of hospital admission, repeated every 6 months	Yes - The SDOH survey was integrated into the electronic medical record
288	23304335	Social and Behavioral History Information in Public Health Datasets	Not applicable	BRFSS, NHANES, NHIS	Alcohol use, drug use, occupation, tobacco use, and other social/behavioral factors	Survey data from BRFSS, NHANES, and NHIS public health surveys	BRFSS - annual; NHANES - every 2 years; NHIS - annual	No - The study analyzed survey data, not EHR data
290	31199345	Incorporating Social Determinants of Health in Electronic Health Records: Qualitative Study of Current Practices among Top Vendors	Not applicable	Screening tools (e.g. PRAPARE), population health management tools, referral management tools, analytic tools	Various SDoH domains	Structured data capture, free text	One-time or periodic screening	Yes - The study examined how EHR vendors are enabling SDoH data capture and use
293	31329894	Psychosocial Information Use for Clinical Decisions in Diabetes Care	Multiple institutions	Not specified	Financial strain, mental health status, life stressors, food security, health literacy, social support	Physician interviews and online survey	One-time data collection	Not specified
305	29854249	Residence, Living Situation, and Living Conditions Information Documentation in Clinical Practice	University of Minnesota, Allina Health	General Architecture for Text Engineering (GATE)	Residence, Living Situation, Living Conditions	Unstructured clinical notes	Not specified	Yes
309	35788378	New Data Resources, Linkages, and Infrastructure for Cancer Health Economics Research: Main Topics from a Panel Discussion	National Cancer Institute	SEER-CMS, SEER-Medicare, SEER-Medicaid	Area-level measures such as median household income, education level, racial/ethnic makeup of population. Plans to incorporate more area-level and individual-level social determinants of health.	Linking cancer registry data (SEER) to CMS administrative data (Medicare and Medicaid enrollment and claims data)	SEER data submitted yearly. SEER-CMS linkage done every other year.	Yes - investigating mechanisms to incorporate more social determinants of health measures into SEER-CMS data.

315	35275090	Linking Electronic Health Records and in-Depth Interviews to Inform Efforts to Integrate Social Determinants of Health into Health Care Delivery: Protocol for a Qualitative Research Study	Geisinger	N/A	Education, financial resource strain, stress, social isolation, transportation challenges, childhood trauma, housing instability	In-depth patient interviews; Extraction of discrete data (diagnoses, medications, utilization) and free text clinic notes from EHR	One-time in-depth interviews with patients. EHR data extracted from 1996-year of interview (2017-2021)	Yes – study links in-depth interview data with patient EHR data
320	34406088	Operationalizing the Population Health Framework: Clinical Characteristics, Social Context, and the Built Environment	Christiana Care Health System	N/A	Smoking status, residential address, demographic and clinical data	Extraction from Epic EHR	One-time extraction of 1 year of admissions data	Yes
326	32701467	Expanding Data Reporting Capacity of Free and Charitable Clinics: A Quality Improvement Project	University of Alabama at Birmingham	SDOH Data Reporting Toolkit	Employment, housing, exposure to violence, food insecurity, utility needs, transportation, education level	Web-based technical assistance toolkit	16 weeks(the program length)	Yes
					CMS Quality Data Reporting Toolkit	Diabetic and hypertension control, tobacco screening, cancer and depression screening, obesity screening/follow-up	16 weeks	
					EHR Implementation Toolkit	Not Specified	16 weeks	
					Economic stability, education, neighborhood and built environment, social and community context	Not Specified	16 weeks	
331	36111269	Identifying Individual Social Risk Factors Using Unstructured Data in Electronic Health Records and Their Relationship with Adverse Clinical Outcomes	University of Virginia, USA	N/A		Unstructured EHR data analysis	During a 12-month period (July 2018 - June 2019)	Yes
339	28679877	Crowdhealth: Holistic Health Records and Big Data Analytics for Health Policy Making and Personalized Health		CrowdHEALTH	Economic stability, education, neighborhood and built environment, social and community context		Continuous (real-time big data analytics)	Yes
348	33735133	Social and Behavioral Variables in the Electronic Health Record: A Path Forward to Increase Data Quality and Utility	Johns Hopkins Health System	N/A	Social and Behavioral Determinants of Health	Structured and unstructured EHR data, focus groups, interviews	Specific to the study conducted	Yes
359	32924796	Feasibility of Mobile Technology to Identify and Address Patients' Unmet Social Needs in a Primary Care Clinic	Wake Forest School of Medicine	Tablet-based digital health system	Food security, housing, transportation	Tablet-based questionnaire via electronic portal	At selected clinic sessions	Yes

360	32430381	Integrating Community and Clinical Data to Assess Patient Risks with a Population Health Assessment Engine (Phate)	University of Texas Health San Antonio	PHATE	Neighborhood SDH data, Clinical data from EHRs	Integration of neighborhood and clinical data in EHR	Not specified	Yes
361	33119102	Comparison of Community-Level and Patient-Level Social Risk Data in a Network of Community Health Centers	OCHIN Network of Community Health Centers	Not specified	Food insecurity, housing insecurity, financial resource strain	Patient-level social risk screening and community-level data from American Community Survey	Not specified	Yes
362	34666982	Routinely Asking Patients About Income in Primary Care: A Mixed-Methods Study	St Michael's Family Health Team, Toronto, Canada	Not specified	Income	Sociodemographic survey including questions about income	Routinely administered	Yes
364	21836738	Incorporating Geospatial Capacity within Clinical Data Systems to Address Social Determinants of Health	Indiana University-Purdue University Indianapolis, School of Liberal Arts, The Polis Center, Indiana University, School of Medicine, Regenstrief Institute, Inc.	Indiana Network for Patient Care (INPC), SAVI Community Information System (SAVI)	Geospatial location of patients, community-level characteristics (e.g., SES, crime incidence, health facility locations)	Integration of EHR data with CIS data, including geocoding and linking with community datasets	Ongoing as part of routine healthcare provision and CIS updates	Yes
369	29930967	Integrating Social Determinants of Health into Primary Care Clinical and Informational Workflow During Care Transitions	University at Buffalo, Elmwood Health Center, University of Minnesota, among others	Coordinating Transitions (CT) Project	Social support networks, home and neighborhood environment, health literacy and well-being, economic forces	Use of electronic health records, care transition alerts, social determinants of health (SDH) assessments, data from Medicaid Data [illegible]	Real-time alerts for recent hospital discharges, ongoing assessment and data collection	Yes
388	32734157	A Case Study of the 1115 Waiver Using Population Health Informatics to Address Disparities		University of California San Diego Health and others,1115 Medicaid Waiver, PRIME program	Race, Ethnicity, Language, Sexual Orientation, Gender Identity	Electronic Health Records, Patient Portal, Clinical Decision Support Tools	Ongoing data collection and analysis, regular updates	Yes
394	27170802	Community Vital Signs: Taking the Pulse of the Community While Caring for Patients	Various institutions including University of California San Diego Health	Community Vital Signs	Socioeconomic and health behavior data, neighborhood factors (poverty level, education, employment status, etc.)	Linkage of aggregated population health data with patient addresses in EHRs, Geocoding technologies	Data is aggregated and updated continuously	Yes

395	36696168	Recording of Social Determinants in Computerized Medical Records in Primary Care Consultations: Quasi-Experimental Study	Institut Català de la Salut, Central Catalonia Territorial Management, Spain	e-cap program, ICD-11 Z-coding	Unemployment, Stressful work schedule, Problems related to living alone, Difficulty in acculturation, Specific problems related to the immediate environment	Coding of social determinants in computerized medical records through ICD-11 Z-coding; Checklist integrated in primary care workflow	Ongoing, with initial 6-month intervention period for the study	Yes, integrated with the e-cap program in primary care settings
396	28103072	Community Health Records: Establishing a Systematic Approach to Improving Social and Physical Determinants of Health	Not Specified	Community Health Records (CHR)	Broad range of community health metrics including social, physical, and lifestyle determinants	Digital forms, linking/mapping external datasets (not specified)	Not Specified	Yes
397	22511197	Identifying Social Risk Via a Clinical Social History Embedded in the Electronic Health Record	Cincinnati Children's Hospital Medical Center	EHR-embedded Social History Template	Financial stability, food scarcity, public benefits, housing conditions, personal safety, parental depression	EHR-embedded template	Not specified	Yes
405	29888079	Evaluation of Flowsheet Documentation in the Electronic Health Record for Residence, Living Situation, and Living Conditions	University of Minnesota, Minneapolis, MN; University of Kansas Medical Center, Kansas City, KS; Brown University, Providence, RI	Flowsheet in EHR	Residence, Living Situation, Living Conditions	Flowsheet data in EHR, including structured and unstructured data	Data from inpatients in 2013	Yes
417	26174867	Community Vital Signs: Incorporating Geocoded Social Determinants into Electronic Records to Promote Patient and Population Health	Oregon Health & Science University; American Medical Informatics Association	Community Vital Signs (Community VS)	Social, economic, occupational, environmental factors	Geocoded community-level data from publicly available sources, integrated into EHRs	Not specified	Yes
420	29190680	Identifying Social Factors Amongst Older Individuals in Linked Electronic Health Records: An Assessment in a Population Based Study	CRPD data	Clinical Practice Research Datalink (CPRD) linked to hospitalisation and deprivation data	Religion, ethnicity, immigration status, small area-level deprivation, place of residence, marital status, living arrangements	Primary care data from CPRD linked to hospitalisation and deprivation data in a cross-sectional study	Not specified in the extract	Yes
432	34842542	Developing the Total Health Profile, a Generalizable Unified Set of Multimorbidity Risk Scores Derived from Machine Learning for Broad Patient Populations: Retrospective Cohort Study	Not specified	Total Health Profile (THP)	Socio-demographic data (age, gender, income, race, ethnicity, etc.), Diagnosis codes, Lab results, Prescriptions, Medical procedural data	Electronic Health Records (EHR)	Retrospective analysis from January 1, 2016, to December 31, 2019	Yes, heavily reliant on EHR data for model development and validation

434	31285969	Adding Social Determinants in the Electronic Health Record in Clinical Care in Hawai'i: Supporting Community-Clinical Linkages in Patient Care	Kaiser Permanente Hawai‘i; Hawai‘i Pacific Health; The Queen’s Health Systems	SWITCH program; SBD measures in EHR; CMS Accountable Health Communities’ Health-Related Social Needs Screening Tool	Social challenges, interpersonal needs, social support, inpatient mobility, housing instability, food insecurity, transportation needs, utility needs, interpersonal safety	EHR integration, social needs screening tools, data fields in EHR, AI-based prediction tools	Not explicitly mentioned	Yes
441	35917481	Issues with Variability in Electronic Health Record Data About Race and Ethnicity: Descriptive Analysis of the National Covid Cohort Collaborative Data Enclave	National COVID Cohort Collaborative (N3C)	N3C Data Enclave	Race and Ethnicity	Electronic Health Records, Standardized Mapping and Harmonization	Updated Weekly	Yes
448	33190565	Required Data Elements for Interprofessional Rounds through the Lens of Multiple Professions	Academic 465-bed tertiary care hospital in a large urban Midwestern city, USA	Interprofessional Rounding Tool	Discharge-related, social determinants of health, hospital safety, nutrition, interprofessional situation awareness, patient history	Semi-structured interviews, Cognitive Task Analysis	Not specified	Yes
462	32577152	The Population Health Outcomes and Information Exchange (Phoenix) Program - a Transformative Approach to Reduce the Burden of Chronic Disease	Wayne State University, Detroit Health Department	PHOENIX Program	Clinical and social determinants of health data	Electronic Health Records, State of Michigan Vital Statistics, US Census/American Communities Survey, Uniform Crime Reports, WSU Investigator Databases, National Center for Education Statistics, Patient/Resident Generated Health Data, and others	Continuously updated, specific frequency not mentioned	Yes
467	37099562	Target-Based Fusion Using Social Determinants of Health to Enhance Suicide Prediction with Electronic Health Records	University of Connecticut; Weill Cornell Medicine; UConn Health	Data Fusion Framework using Add Health Data	Social determinants of health (e.g., social support, parental relationship quality, peer interactions, perceptions of town safety, etc.)	Fusion of EHR data from Connecticut’s Hospital Inpatient Discharge Database and survey data from The National Longitudinal Study of Adolescent to Adult Health (Add Health)	Retrospective study (Data collected from 2012 to 2017 for the HIDD and baseline wave in 1994 for Add Health)	Yes

471	35758723	Addressing Social Needs in the Clinical Setting: Description of Needs Identified in a Quality Improvement Pilot across 3 Community Hospital Service Areas	Advocate Aurora Health	NowPow, a Unite Us Company Platform	Financial stability, food and nutrition, transportation, housing, employment	Digital platform for screening and referral to community resources, integrated with EHR	Data collected from November 2018 to November 2020	Yes
473	30715327	Incorporating a Location-Based Socioeconomic Index into a De-Identified I2b2 Clinical Data Warehouse	University of Nebraska Medical Center	Informatics for Integrating Biology and the Bedside (i2b2)	Socioeconomic variables from the American Community Survey (ACS) including education, employment, poverty, public assistance, family structure, income	Integration of EHR data with ACS data, geocoding patient data	Data extracted from the EHR and ACS for the period 2011-2015	Yes
478	29029685	Using Structured and Unstructured Data to Identify Patients' Need for Services That Address the Social Determinants of Health	Indiana University Richard M. Fairbanks School of Public Health, Regenstrief Institute, Indiana University School of Medicine, Eskenazi Health	Not specified	Social work, behavioral health, nutrition counseling, respiratory therapy, financial planning, medical-legal partnership assistance, patient navigation, pharmacist consultation	Structured billing and scheduling data, and unstructured EHR data	Cross-sectional study period from April 2012 to July 2016	Yes
480	25092009	Envisioning a Social-Health Information Exchange as a Platform to Support a Patient-Centered Medical Neighborhood: A Feasibility Study	University of Texas Southwestern Medical Center, Parkland Center for Clinical Innovation	Social-Health Information Exchange (S-HIE)	Homelessness, poverty, food insecurity, social isolation, etc.	Web-based IT infrastructure, interviews, and town hall meetings	Study period from October 2010 to May 2011	Yes
482	37051408	Evaluating Demographic Data to Improve Confidence in Equity Analytics in a Children's Hospital	Akron Children’s Hospital	Epic Systems (EHR)	Race, Sex, Ethnicity, Language, Zip Code, Payer, Age	Electronic Health Record (EHR), Training for REaL (Race, Ethnicity, and Language) Data Collection	Not Specified	Yes
500	36973053	Patient-Reported Social Risks and Clinician Decision Making: Results of a Clinician Survey in Primary Care Community Health Centers	OCHIN Inc, University of California San Francisco, Kaiser Center for Health Research	EHR-embedded card study	Not specified in the extract	Embedded EHR surveys	During a 3-week period (daily prompts for clinicians)	Yes
508	27834242	Communityrx: A Population Health Improvement Innovation That Connects Clinics to Communities	University of Chicago, Community Health Centers	CommunityRx	37 prevalent social and medical conditions	E-prescribing model with community engagement, youth workforce, community health information specialists	Not specified in the extract	Yes

514	26969124	Utility of Linking Primary Care Electronic Medical Records with Canadian Census Data to Study the Determinants of Chronic Disease: An Example Based on Socioeconomic Status and Obesity	Queen's University-Canada	CPCSSN	Socio-economic status, BMI, Obesity, Area-level deprivation	EMR Data, Census Data	January 1st to December 31st, 2011 (One-time extraction)	Yes
519	22511015	Harmonized Patient-Reported Data Elements in the Electronic Health Record: Supporting Meaningful Use by Primary Care Action on Health Behaviors and Key Psychosocial Factors	Multiple Institutions including Virginia Tech, National Institutes of Health, Harvard University, National Cancer Institute, Virginia Commonwealth [illegible]	EHR Harmonized Patient-Reported Data Elements	Health behaviors (e.g., exercise), psychosocial issues (e.g., distress), patient-centered factors (e.g., demographics)	Electronic Health Records (EHR), Surveys	Frequency varies: single assessment (e.g., demographics), annual assessment (e.g., health behaviors), or more frequent (e.g., patient goals)	Yes
527	30691480	Study Protocol: A Pragmatic, Stepped-Wedge Trial of Tailored Support for Implementing Social Determinants of Health Documentation/Action in Community Health Centers, with Realist Evaluation	Kaiser Permanente–Center for Health Research, OCHIN, Inc	OCHIN: EHR-based Social Determinants of Health Documentation	Various social determinants of health (e.g., chronic stress, poverty, lack of access to healthy foods/safe exercise)	Electronic Health Records (EHR), Patient Portals, Tablets at Clinic, Paper-based Entry	Ongoing, as part of routine care	Yes
529	28269967	Representing Residence, Living Situation, and Living Conditions: An Evaluation of Terminologies, Standards, Guidelines, and Measures/Surveys	Institute for Health Informatics, University of Minnesota; Allina Health, Minneapolis, MN; Center for Biomedical Informatics, Brown University	Various (including SNOMED-CT, US Census, HL7, openEHR, etc.)	Residence, Living Situation, Living Conditions	Electronic Health Records (EHR), Surveys, Standards, and Guidelines	Variable (based on source standards and guidelines)	Yes
531	34803048	The Direct Clinic-Level Cost of the Implementation and Use of a Protocol to Assess and Address Social Needs in Diverse Community Health Center Primary Care Clinical Settings	Duke University School of Medicine; University of North Carolina at Chapel Hill; Lincoln Community Health Center; Durham VA Healthcare [illegible]	PRAPARE (Protocol for Responding to and Assessing Patients' Assets, Risks, and Experiences)	Food insecurity, housing instability, social isolation, unemployment, and other social determinants	Interviews, Activity-Based Costing Tool	Ongoing maintenance after initial implementation	Yes

536	34623308	Using Electronic Health Record-Based Clinical Decision Support to Provide Social Risk-Informed Care in Community Health Centers: Protocol for the Design and Assessment of a Clinical Decision Support Tool	OCHIN member CHCs	EHR-based CDS tools	Various social risks (e.g., food, housing, transportation insecurity)	EHR tools, patient-reported data	Not specified	Yes
539	30354371	Compass-Cp: An Electronic Application to Capture Patient-Reported Outcomes to Develop Actionable Stroke and Transient Ischemic Attack Care Plans	Wake Forest University Health Sciences, 20 North Carolina hospitals	COMPASS-CP	Physical, mental, and social well-being; medication adherence; financial challenges; caregiver health and needs	Web-based application, iPad application	At 2-day post-discharge and 7-14 day clinic visit	Yes
559	36375772	Methods for Development and Application of Data Standards in an Ontology-Driven Information Model for Measuring, Managing, and Computing Social Determinants of Health for Individuals, Households, and Communities Evaluated through an Example of Asthma	Department of Population Health, Dell Medical School, University of Texas at Austin	Asthma CDS tools	Environmental factors important for managing pediatric asthma, including local outdoor air quality monitors, indoor air quality questionnaire data	Questionnaire, Local outdoor air quality monitors, EHR data	Not specified in the excerpt	Yes
560	30202181	The Community and Patient Partnered Research Network (Cpprn): Application of Patient-Centered Outcomes Research to Promote Behavioral Health Equity	Community effort	CPPRN	Behavioral health and social risk factors	Community partnered participatory research (CPPR), large, de-identified dataset across county agencies, information technology-supported screening	Not specified	Yes
575	31083298	The Association of Black Cardiologists (Abc) Cardiovascular Implementation Study (Cvis): A Research Registry Integrating Social Determinants to Support Care for Underserved Patients	Morehouse School of Medicine, Duke University School of Medicine, Association of Black Cardiologists, Others	ABC Cardiovascular Implementation Study (CVIS)	Socio-demographic data, clinical and biospecimen data, social determinants of health	Prospective cohort registry; electronic data capture, mobile health technology, and APIs to health system or practice EHRs	Ongoing, regular data collection as standard of care dictates; approximately every 6 months	Yes
593	22937207	The Murdock Study: A Long-Term Initiative for Disease Reclassification through Advanced Biomarker Discovery and Integration with Electronic Health Records	Duke Translational Medicine Institute	MURDOCK Study	Not explicitly mentioned	Biospecimens, EHR data, environmental and social data	Annually (for registry updates)	Yes

600	1458861	The Chronic Disease Data Bank Model: A Conceptual Framework for the Computer-Based Medical Record	Not explicitly stated	ARAMIS, ATHOS	Chronic diseases (e.g., rheumatic diseases, cardiovascular disease, pulmonary disease, renal disease, neurological disease, HIV-associated disease)	Time-oriented data, longitudinal data collection, Health Assessment Questionnaire (HAQ)	Every 6 months (as indicated for ARAMIS)	Yes
605	37008604	Insights into Measuring Health Disparities Using Electronic Health Records from a Statewide Network of Health Systems: A Case Study	University of Wisconsin School of Medicine and Public Health, Madison, WI, USA	Wisconsin Collaborative for Healthcare Quality (WCHQ) data repository	Health disparities (race and ethnicity, insurance status and type, geographic disparity)	EHR data collection, common data model, structured patient-level data (demographics, encounters, hospitalizations, medications, laboratory values, clinical values, smoking history, providers, and clinics)	Not explicitly stated	OMOP
626	36711509	Social Determinants of Health: A Need for Evidence-Based Guidelines on How to Capture Data on Underserved Patients	Mayo Clinic	Not specified	Housing instability, lack of transportation, financial concerns, social isolation	Electronic medical record data collection, patient questionnaire	Annually (as part of clinical care)	Yes
629	35939597	A Need for Digitally Inclusive Health Care Service in the United States: Recommendations for Clinicians and Health Care Systems	Mayo Clinic Health System–Northwest Wisconsin Region, Eau Claire, WI, USA; Mayo Clinic, Rochester, MN, USA	Not specified	Digital access (broadband internet connectivity and access to smart devices), digital health literacy	Electronic health record screening for digital access and literacy	At primary visit and subsequent visits	Yes
644	31659920	An Evidence-Based Strategy to Achieve Equivalency and Interoperability for Social-Behavioral Determinants of Health Assessment, Storage, Exchange, and Use	Cerner Corporation, USA; University of Kansas, USA	PRAPARE (Protocol for Responding to and Assessing Patients' Assets, Risks, and Experiences) tool and IOM instrument	Various social-behavioral determinants of health	Three-tier scoring strategy for data equivalence	Not specified	Yes
692	35658093	Covid-19 Variant Surveillance and Social Determinants in Central Massachusetts: Development Study	Medical School, Worcester, MA, United States	University of Massachusetts' Graphical user interface for Geographic Information (MAGGI)	Race, ethnicity, median family income, housing, language, poverty, population density, social vulnerability index	Integrated data from electronic medical records, SARS-CoV-2 genomic analysis, and public health resources	Monthly	Yes

693	34867537	The Biopsychosocial Model and Perinatal Health Care: Determinants of Perinatal Care in a Community Sample	University of Rochester, Rochester, NY, United States	Kotelchuck Index (KI)	Socio-demographic (race, ethnicity, income, education, marital status), psychological (affective symptoms, trauma experience), social and family context (social support, family size)	Self-report questionnaire, interview, abstracted from electronic medical records	At each trimester visit	Yes
727	35186824	Thrive Conceptual Framework and Study Protocol: A Community-Partnered Longitudinal Multi-Cohort Study to Promote Child and Youth Thriving, Health Equity, and Community Strength	University of Pittsburgh, PA, United States	Various (including PROMIS, NSCH, YRBS)	Demographics, family relationships, safety, vibrant communities, healthy environments, racial justice, equity and inclusion, overall health, physical activity, eating/nutrition, height and weight, sleep, screen time, health behaviors, alcohol, tobacco, and drug use, sexual activity, child mental health, academic performance	Analysis of electronic health records, school records, health and human services use, and other administrative secondary data combined with parent/caregiver and youth-reported survey data.	Longitudinal, spanning from pregnancy through adolescence	Yes
733	32811587	Stability of Food Insecurity Status in Paediatric Primary Care	St. Christopher's Hospital for Children, Philadelphia, PA, USA	Hunger Vital Sign screener	Food insecurity	Electronic health records	Screened at intervals of 0-6 months, 6-12 months, and more than a year	Yes

634	33209839	Comparing the Performance of Two Social Risk Screening Tools in a Vulnerable Subpopulation	Social risks, behavioral health, stigma, functioning, self-rated health, concerns about healthcare costs, trust in healthcare providers	48 questions (social risk screening: AHC, YCLS)	Home-grown	KPSC members	Telephone screening	Active and Passive	Personal and structural	both	
637	33425666	Quantitative and Qualitative Results from Implementation of a Two-Item Food Insecurity Screening Tool in Healthcare Settings in Connecticut	Food	2 screening questions	Home-grown	Patients who presented for any type of visit	A federally qualified health center (CSHHC)	Active	Personal	personal	
695	32974614	Protocol for Comparing Two Training Approaches for Primary Care Professionals Implementing the Safe Environment for Every Kid (Seek) Model	Parental depression and major stress, substance abuse, IPV, harsh discipline, and food insecurity	SEEKonline (SEEK Parent Questionnaire-R)	Not specified	Children's parent	Primary care settings, with a focus on pediatric and family medicine practices	Active	Personal	personal	
778	34930500	Using a Participatory Method to Test a Strategy Supporting the Implementation of a State Policy on Screening Children for Adverse Childhood Experiences (Aces) in a Federally Qualified Health Center System: A Stepped-Wedge Cluster Randomized Trial	Not specific	PSC	Home-grown	Caregivers of children aged 0–5 years in the FQHC system	Child primary care visits (5 clinics)	Active and Passive	Personal	personal	
368	34930500	Part I: A Quantitative Study of Social Risk Screening Acceptability in Patients and Caregivers	Social risks (housing stability, food security, transportation, utilities, personal safety)	Center for Medicare and Medicaid Innovation Accountable Health	Home-grown	Adult patients and caregivers of pediatric patients	Primary care clinics and emergency departments across 9 states	Active	personal and structural	both	Added from Cat2
656	35978539	Implementing Health Related Social Needs Screening in an Outpatient Clinic	Housing quality and security, financial need, food insecurity, health literacy, immigration needs, transportation, utilities, domestic violence, social isolation	HRSN Screening Tool	Home-grown	Patients of a FQHC, including those with chronic diseases and frequent clinic visits	Outpatient clinic setting within a FQHC	Active	Both personal (health literacy, immigration needs) and structural determinants (housing quality, food	both	
148	35308947	Extracting Patient-Level Social Determinants of Health into the Omop Common Data Model	Housing Insecurity/Instability/Homelessness, Food insecurity, Employment, Education, Health Care/Medicine Access & Affordability, Immigration/Migrant Status/Refugee Status, Household size, Annual Household Income	Not specified in the abstract(OMOP -SDoH extraction flowsheet)	Home-grown	Patients tested for COVID-19 within UW Medicine Health Network	Health Network Enterprise Data Warehouse (EDW), including various hospitals and outpatient clinics	Active (via Epic Clarity FlowSheet structure)	Personal	personal	
465	27170792	Building a Foundation to Reduce Health Inequities: Routine Collection of Sociodemographic Data in Primary Care	Sociodemographic Data (14 questions covering social determinants of health)	Not specified	Home-grown	Adult patients in primary care	Family medicine clinic, St. Michael's Hospital, Toronto	Active (self-administered using an electronic tablet)	Both Personal and Structural	both	
512	31376277	Assessing the Availability of Data on Social and Behavioral Determinants in Structured and Unstructured Electronic Health Records: A Retrospective Analysis of a Multilevel Health Care System	Patient address/zip code, Ethnicity, Race, Preferred language, Alcohol use, Smoking status, Social connection/isolation, Housing issues, Income/Financial resource strain	N/A (Study-based Analysis)	Home-grown	Patients within a multilevel academic health care system	Various clinical settings (e.g., inpatient and outpatient)	Passive (Analysis of EHR data)	Both personal (e.g., alcohol use, smoking status) and structural (e.g., housing issues, income/financial resource strain)	both	
516	36036781	Implementing a Process for Screening Hospitalized Adults for Food Insecurity at a Tertiary Care Center	Economic Stability	USDA's HFSS-based 2-item Screen	Vendor-specific (Epic System)	Hospitalized Adults	26-bed Adult Hospital Medicine Unit	Active	Personal	personal	
646	36161115	Addressing Social Risk Factors in the Inpatient Setting: Initial Findings from a Screening and Referral Pilot at an Urban Safety-Net Academic Medical Center in Virginia, USA	Economic Stability, Housing Stability, Transportation	Health Leads (based on)	Home-grown	Hospitalized Patients	Internal Medicine Unit at VCU Health	Active	Structural	structural	
656	35978539	Implementing Health Related Social Needs Screening in an Outpatient Clinic	Housing, Financial, Food Insecurity, Health Literacy, Immigration, Transportation, Utilities, Domestic Violence, Social Isolation	HRSN Screening Tool	Home-grown	FQHC Patients	Outpatient Clinics of a FQHC	Active	Structural	structural	

EndNote_ID	PubMed_ID	TItle	Apprach	features	Accuracy(performance)	Incorporate with EHR	NLP Tool/System	Data Source 1	Data Source 2	Data Source 3
203	32131117	Detecting Social and Behavioral Determinants of Health with Structured and Free-Text Clinical Data	Combination of machine learning and NLP techniques	Bag-of-words, TF-IDF weights, structured EHR data	Variable; e.g., LGBT status F1 = 82.7, IV drug use F1 = 28.5	Yes	Not specified	Columbia University Medical Center		
440	30815082	Towards the Inference of Social and Behavioral Determinants of Sexual Health: Development of a Gold-Standard Corpus with Semi-Supervised Learning	Machine learning	tf-idf weights, lexical features, ontological features from UMLS	Poor performance on individual SBDH labels	Yes	In-house NER system	Columbia University Medical Center		
201	35042703	Simplified Data Science Approach to Extract Social and Behavioural Determinants: A Retrospective Chart Review	Machine learning - Naive Bayes, SVM, logistic regression, random forest	Bag-of-words, n-grams, custom regex for negatives	Housing: 92.18%, Tobacco: 84.68%, Alcohol: 82.79%	Yes	Not specified	Harborview Medical Center		
176	35005628	Mimic-Sbdh: A Dataset for Social and Behavioral Determinants of Health	Machine Learning (Random Forest, XGBoost, Bio-ClinicalBERT)	Classification of EHR notes for 7 SBDHs, marking of SBDH-related keywords	F1 score range from 0.69 (Drug Use) to 0.96 (Community-Present)	No	Not specified	MIMIC Dataset		
704	36652267	Evaluation of a Model to Target High-Risk Psychiatric Inpatients for an Intensive Postdischarge Suicide Prevention Intervention	Machine learning (Super Learner ensemble)	NLP extracted terms/topics, geocoded data, EHR data	AUROC 0.747-0.780, net benefit at multiple thresholds	Yes	Custom NLP methods	VA EHR and databases		
227	29084046	Using Clinical Notes and Natural Language Processing for Automated Hiv Risk Assessment	Machine learning (supervised,Random Forest, NLP techniques including TF-IDF and LDA	Random Forest, NLP techniques including TF-IDF and LDA	F-measures: 0.59 for baseline model, 0.63 for baseline + NLP topic model, 0.74 for baseline + NLP keyword model	Yes	Not specified	New York Presbyterian Hospital, Columbia University Medical Center		
312	34984989	Evaluation of a Natural Language Processing Approach to Identify Social Determinants of Health in Electronic Health Records in a Diverse Community Cohort	Machine learning (supervised)	n-grams, tf-idf	Housing: Sensitivity = 0.67, Specificity = 0.89, κ = 0.51; Medical Care: Sensitivity = 0.55, Specificity = 0.73, κ = 0.20; Transportation: Sensitivity = 0.79, Specificity = 0.87, κ = 0.58; Combined SDoH: Sensitivity = 0.77, Specificity = 0.69, κ = 0.45	No	spaCy	Kaiser Permanente Mid-Atlantic States (KPMAS)		
223	34514351	Identification of Social Determinants of Health Using Multi-Label Classification of Electronic Health Record Clinical Notes	Multi-label Learning (MLL) with various ML models,BI-LSTM, XGBoost, SVM, KNN, Random Forest	Clinical note sentences, tokenization, stemming, lemmatization	AUC-ROC: 93.9, AP: 0.76, Hamming loss: 0.12	Yes	Not specified	University of North Carolina Health System		
166	31095055	Measuring Exposure to Incarceration Using the Electronic Health Record	Natural language processing (NLP) and machine learning	Bag-of-words model with stop word removal. Support vector machine classifier.	Sensitivity: 63.5%, Specificity: 95.9%, F1-score: 0.75, Matthews correlation coefficient: 0.64	No	Yale cTAKES extension (YTEX)	Yale University School of Medicine		

183	28295260	Hospital Readmission and Social Risk Factors Identified from Physician Notes	MTERMS(Rule-based system)	Lexicon-based information extraction	Not reported	Yes	MTERMS NLP System	Partners Healthcare System	
363	29295142	Assessing the Representation of Occupation Information in Free-Text Clinical Documents across Multiple Sources	Not specified	Not specified	Not reported	No	Rule-based		The study analyzes representation of occupation information in free-text clinical notes from multiple sources.
336	35308929	Creation of a Mapped, Machine-Readable Taxonomy to Facilitate Extraction of Social Determinants of Health Data from Electronic Health Records	Rule-based	Domain-specific taxonomy, keyword searches	N/A	Yes	N/A	Veradigm EHR database	
340	35533516	A Case for Developing Domain-Specific Vocabularies for Extracting Suicide Factors from Healthcare Notes	Rule-based	Domain-specific lexicons, word2vec for lexicon expansion	Higher recall than structured data, improved concentration of risk factors in top predicted suicide risk patients	Yes	CLEVER	VA Corporate Data Warehouse	
668	30975223	Moonstone: A Novel Natural Language Processing System for Inferring Social Risk from Clinical Narratives	Rule-based	Semantic grammar, inference rules, type hierarchy	Precision: 0.66-0.98, Recall: 0.75-0.97, Accuracy: 0.63-0.95	No	Moonstone	Veterans Health Administration notes	
746	35658116	Prediction of Future Health Care Utilization through Note-Extracted Psychosocial Factors	Rule-based	Regular expressions, word2vec	Precision >0.9	Yes	Custom rule-based system	Oregon Health & Science University EHR	
422	33766838	Text Mining Occupations from the Mental Health Electronic Health Record: A Natural Language Processing Approach Using Records from the Clinical Record Interactive Search (Cris) Platform in South London, Uk	Rule-based and machine learning	Conditional random field classifier, support vector machine classifier	Precision: 0.77-0.79, Recall: 0.77-0.79	Yes	Developed on GATE platform	Clinical Record Interactive Search (CRIS) case register	
635	35265945	Comparative Analysis of Patient Distress in Opioid Treatment Programs Using Natural Language Processing	Rule-based and machine learning	Custom lexicons, entity extraction	N/A	Yes	CLAMP	Mount Sinai Health System OTP database	
252	32308819	Determination of Marital Status of Patients from Structured and Unstructured Electronic Healthcare Data	Rule-based NLP	N-grams, Vocabulary Mapping, Context Detection, Document Inference	NLP Engine: F1 Score 0.97 (document-level), 0.95 (patient-level); Machine Learning: F1 Score 0.63	Yes	EasyCIE	University of Utah Health Sciences Center	

321	35224458	Development and Assessment of a Natural Language Processing Model to Identify Residential Instability in Electronic Health Records' Unstructured Data: A Comparison of 3 Integrated Healthcare Delivery Systems	Rule-based NLP	Expert-developed patterns, vocabulary mapping, negation handling	PPV: 0.45-1.00, Sensitivity: 0.68-0.96, Specificity: 0.69-1.00	Yes	EntityRuler module of spaCy 2.3 Python toolkit	Johns Hopkins Health System (JHHS), Kaiser Permanente Mid-Atlantic States (KPMAS), KP Southern California (KPSC)		
459	34474188	Rehoused: A Novel Measurement of Veteran Housing Stability Using Natural Language Processing	Rule-based NLP system	Defined entity classes related to homelessness and stable housing. Used semantic and syntactic patterns for entity extraction.	Moderate document classification performance (F1 0.658). Patient-level classification aligned well with reference standard data.	Yes	Custom rule-based system in medspaCy	US Department of Veterans Affairs		
297	28807893	Optimizing the Use of Electronic Health Records to Identify High-Risk Psychosocial Determinants of Health	Rule-based NLP, Word Recognition Software	Natural Language Processing, Term Indexing, Data Aggregation, Negation Detection, Date Detection	Precision: 0.80, Recall: 0.98, Balanced F-measure: 0.88	Yes	QPID (Queriable Patient Inference Dossier)	Massachusetts General Hospital		
313	37128364	An Investigation of the Representation of Social Determinants of Health in the Umls	Rule-based NLP，clinicalBERT-based classification, ML classifiers (LR, RF, K-Nearst)	Dictionaries, semantic types	Extracted 198,677 SDoH concepts, 91% classification accuracy	No	Clinical BERT, EASE	UMLS, Veteran Health Administration's EHR data		
442	34513783	Measuring the Value of a Practical Text Mining Approach to Identify Patients with Housing Issues in the Free-Text Notes in Electronic Health Record: Findings of a Retrospective Cohort Study	Rule-based text mining using regular expressions (RegEx)	Developed linguistic patterns and phrases for 5 categories of housing issues	High precision (96-95-94% at phrase, note, patient level), low recall (30-32-41% at phrase, note, patient level)	Yes	Custom RegEx patterns	Large multispecialty medical group in New England, USA		
246	35309014	A Study of Social and Behavioral Determinants of Health in Lung Cancer Patients Using Transformers-Based Natural Language Processing Models	Supervised deep learning - (Transformers: BERT, RoBERTa)	(Transformers: BERT, RoBERTa)	Best F1-score: 0.8791 (strict), 0.8999 (lenient) for BERT_general	Yes	not specified	University of Florida Health Integrated Data Repository		
181	36560764	Clinical Application of Detecting Covid-19 Risks: A Natural Language Processing Approach	Supervised deep learning - BiLSTM-CNN-CRF model with transformer-based embeddings	BiLSTM, CNN, CRF layers; Transformer embeddings	Micro-F1: 0.949, Macro-F1: 0.9537	No	Not specified	MIMIC Dataset	PubMed	LitCOVID

175	35007754	Classifying Social Determinants of Health from Unstructured Electronic Health Records Using Deep Learning-Based Natural Language Processing	Supervised deep learning - CNN, LSTM, BERT	CNN: Convolutional layers, max pooling, dropout regularization; LSTM: Recurrent layers, dropout regularization; BERT: Pre-trained transformer layers	BERT: Micro-F1 0.69, Macro-AUC 0.907	No	Not specified	MIMIC Dataset	
468	37011635	A Marker-Based Neural Network System for Extracting Social Determinants of Health	Supervised deep learning - Multi-stage pipeline with marker-based NER, relation classification, and text classification	Novel marker-based NER model, contextualized embeddings (BERT)	F1 of 0.9101, 0.8053, and 0.9025 on N2C2 dataset. State-of-the-art compared to other shared task systems.	No,	Custom pipeline	MIMIC Dataset	UW Harborview Medical Center
164	35419333	Assessing the Documentation of Social Determinants of Health for Lung Cancer Patients in Clinical Narratives	Supervised deep learning - Transformer-based NLP model using BERT architecture	Masked language modeling	F1-score 0.9216	No	BERT-based NLP pipeline	University of Florida Health (UF Health) IDR	
612	34747714	Risk Factors Associated with Nonfatal Opioid Overdose Leading to Intensive Care Unit Admission: A Cross-Sectional Study	Supervised deep learning (BERT)	Word-level annotations	99.82% of SBDH from NLP vs 0.18% from structured data	Yes	BERT	MIMIC Dataset	
645	37002953	Extracting Social Determinants of Health Events with Transformer-Based Multitask, Multilabel Named Entity Recognition	Supervised deep learning (BioClinicalBERT)	2-stage architecture, multi-label NER	0.87 precision, 0.89 recall, 0.88 F1	No	Custom model based on BioClinicalBERT	n2c2 Track 2 competition dataset	
520	37130345	Leveraging Natural Language Processing to Augment Structured Social Determinants of Health Data in the Electronic Health Record	Supervised deep learning (mSpERT model)	Contextualized embeddings (BERT), multi-task learning	0.86 F1 on test set, 0.77-0.86 F1 on validation notes	Yes	mSpERT model	University of Washington Medicine	
484	36920391	Associations between Natural Language Processing-Enriched Social Determinants of Health and Suicide Death among Us Veterans	Supervised deep learning (RoBERTa model)	Contextualized word embeddings, multi-task learning framework	No accuracy metrics reported	Yes	Custom RoBERTa model	US Veterans Health Administration	
672	33791684	Automated Coding of under-Studied Medical Concept Domains: Linking Physical Activity Reports to the International Classification of Functioning, Disability, and Health	Supervised deep learning (SVM, KNN, DNN) and deep learning (BERT)	Word embeddings, ICF code definitions	84% macro F1 score	No	Custom implementations	Physical therapy notes from NIH Clinical Center	
398	31438179	Identifying Patients with Significant Problems Related to Social Determinants of Health with Natural Language Processing	Unsupervised learning	Lexical association metrics like $\chi2$ test for query expansion, TF-IDF for document ranking	Precision >0.90 for all concepts except social isolation after iterative improvements (initial precision 0.60-0.68)	Yes	Not specified	Oregon Health & Science University	

501	29016793	Mining 100 Million Notes to Find Homelessness and Adverse Childhood Experiences: 2 Case Studies of Rare and Severe Social Determinants of Health in Electronic Health Records	Unsupervised learning and information retrieval system	word2vec for query expansion, TF-IDF weighted cosine similarity for retrieval	Homelessness: P@185 0.93, AUPRC 0.95. ACE: P@1000 0.70, AUPRC 0.73	No	Custom information retrieval system	Vanderbilt University Medical Center Synthetic Derivative database
274	32570369	Discovering New Social Determinants of Health Concepts from Unstructured Data: Framework and Evaluation	Word Embedding Model, Human-in-the-loop	Word Embedding Techniques, Euclidean Distance for Term Similarity, Skip-gram Model with Negative Sampling, Subsampling	Not explicitly provided (Focused on term discovery and matching, not accuracy)	No	Custom-built model using state-of-the-art embedding techniques	IBM Research, Ireland; NIHR Greater Manchester Patient Safety Translational Research Centre, University of Manchester

EndNote_ID	PubMed_ID	Title	Study types	Assessed bias(Yes/No)	Bias type	Health event	diseases	prevention method	quality measures	definitions(standard/come up)	prediction model/not	Comment
147	36367672	Association between Gentrification and Health and Healthcare Utilization	Retrospective Cohort	No	N/A	Health and Healthcare Utilization	Cardiovascular Disease, Hypertension, Diabetes, Obesity, Asthma, Depression	Not Specified	Multivariable Logistic and Poisson Regression Models	Not Specified	No	
153	35725136	Race Versus Social Determinants of Health in Covid-19 Hospitalization Prediction	Observational Study	No	N/A	COVID-19 Hospitalization	Cardiometabolic Diseases	Not Specified	hospitalization rates	Not Specified	Yes	
154	35420651	Association between Social Determinants of Health and Retinopathy of Prematurity Outcomes	Retrospective Cohort Study	No	N/A	Diagnosis and severity of Retinopathy of Prematurity (ROP)	Retinopathy of Prematurity	Not specified	Outcome measures include diagnosis and severity of ROP, determined by a classification system developed by the Early Treatment for Retinopathy of Prematurity Cooperative Group	Standard (STROBE reporting guidelines, American Academy of Pediatrics guidelines for ROP screening)	No	
158	36475934	Social Determinants of Health and Hypertension Control in Adults with Medicaid	Retrospective Analysis	No	N/A	Hypertension Control	Hypertension	Not specified	Blood pressure measurement, control of hypertension	Standard (United States Department of Agriculture's Adult Food Security Survey Module, Weekly Stress Inventory, etc.)	No	
160	36561097	Social Determinants of Health Influence Early Outcomes Following Lumbar Spine Surgery	Retrospective Observational Study	No	N/A	Postoperative Outcomes (Length of Stay, Discharge Disposition, Readmissions)	Lumbar Spine Surgery	Not specified	Length of stay (LOS), Discharge to skilled nursing facility, 30-day emergency department return, 30-day readmission	Custom (Self-Reported Social Determinants of Health Survey)	No	
162	35864034	Social Determinants of Health Associated with Patient Portal Use in Pediatric Diabetes	Cross-Sectional Study	No	N/A	Patient portal utilization	Type 1 Diabetes	Not specified	Association between SDOH and patient portal use in pediatric diabetes	Custom (ON-Marg, deprivation index used in Canada)	No	
170	35822762	Ethnicity, Social Determinants of Health, and Pediatric Primary Care During the Covid-19 Pandemic	Retrospective Study	No	N/A	Pediatric Primary Care during COVID-19	COVID-19 Impact on Pediatric Healthcare	Not specified	Health outcomes (routine medical care delay, vaccine delays, utilization of acute and ED visits)	Custom (Self-Reported Social Determinants of Health Survey)	No	
172	35060411	Minnesota Electronic Health Record Consortium Covid-19 Project: Informing Pandemic Response through Statewide Collaboration Using Observational Data	Serial Cross-Sectional Study	No	N/A	COVID-19 Pandemic Monitoring	COVID-19	Disease and Syndromic Surveillance	COVID-19 Testing Rates and Positivity	Custom (EHR Data Analysis)	No	
177	36031449	Social Determinants of Health in Pediatric Scald Burns: Is Food Access an Issue?	Retrospective Observational Study	No	N/A	Pediatric scald burns	Scald burns	Not specified	Association between scald burns and various factors, including socioeconomic status, race, and access to food	CDC Social Vulnerability Index	No	
184	33948528	Socioeconomic Determinants of Tertiary Rhinology Care Utilization	Retrospective review of electronic health records	No	-	Utilization of tertiary rhinology care services	Chronic Rhinosinusitis (CRS)	-	Utilization rates, correlation with census data, multivariate regression analyses	Standard (International Classification of Diseases, Ninth and Tenth Revision codes)	No	

187	33537273	Multi-Morbidity in the Older Person: An Examination of Polypharmacy and Socioeconomic Status	Descriptive analysis, literature review	No	-	Managing multi-morbidity in older adults, risk of polypharmacy	Multi-morbidity	Social prescribing, use of EHRs, redistributive welfare policies	-	-	No
191	36653224	Role of Race and Insurance Status in Prostate Cancer Diagnosis-to-Treatment Interval	Retrospective Observational Study	No	N/A	Prostate Cancer Diagnosis-to-Treatment Interval	Prostate Cancer	N/A	the interval between multiparametric MRI (mpMRI)-to-biopsy and biopsy-to-prostatectomy in prostate cancer patients	Not Specified	No
192	36427682	Substance Use Disorders and Social Determinants of Health from Electronic Medical Records Obtained During Kentucky's "Triple Wave"	Retrospective analysis of EMRs	No	-	Substance use disorders in the context of social determinants of health	Opioid Use Disorder (OUD), Stimulant Use Disorder, Polysubstance Use	-	Utilization of healthcare services, housing instability, co-morbid mental and physical health conditions, drug-related events	Standard (ICD-10-CM codes)	No
196	33512511	Determinants of Stage at Diagnosis of Hpv-Related Cancer Including Area Deprivation and Clinical Factors	Retrospective case–control study	No	-	HPV-related cancers	HPV-related cancers (anal, cervical, oropharyngeal, penile, vulvar, vaginal)	-	SEER stage guidelines, Area Deprivation Index (ADI)	Standard (ICD-9, ICD-10, American Community Survey)	No
198	35673163	Using Ehr Data to Identify Social Determinants of Health Affecting Disparities in Cancer Survival	Observational Study	No	N/A	Cancer Survival	Lung Cancer, Colorectal Cancer, Breast Cancer, Multiple Myeloma, Prostate Cancer	N/A	Survival time (short survival <1 year, long survival >5 years), Logistic Regression Analysis	Standard (uses established age groups and race categories)	No
207	25559099	Linking Electronic Health Records with Community-Level Data to Understand Childhood Obesity Risk	Retrospective Observational Study	No	N/A	Assessing childhood obesity risk based on environmental and socioeconomic factors	Childhood obesity	Not specified	Association between the economic hardship index (EHI) and race/ethnicity, age, sex and childhood obesity rates in Wisconsin children using an EHR dataset	Standard (economic hardship index (EHI) and race/ethnicity, age and sex)	No
211	33757936	Social Determinants of Health and Coronavirus Disease 2019 in Pregnancy	Cross-Sectional Study	No	N/A	SARS-CoV-2 Infection and COVID-19 Severity	COVID-19	N/A	Polymerase Chain Reaction Test Positivity, Severity of Illness Categories	Standard (Uses established socioeconomic and demographic categories)	No
215	33359151	Neighborhood Socioeconomic Status and Identification of Patients with Ckd Using Electronic Health Records	Observational Study	No	N/A	Chronic Kidney Disease (CKD) Prevalence	Chronic Kidney Disease (CKD)	N/A	Prevalence of CKD, estimated glomerular filtration rate, proteinuria	Standard (Uses Census tract neighborhood SES measures and individual-level health insurance status)	No
220	30834776	Geospatial, Clinical, and Social Determinants of Hospital Readmissions	Retrospective Cohort Study	No	N/A	90-day readmission	Not specified	N/A	Association between 90-day readmission and geospatial-sociodemographics and clinical characteristics	Standard (American Community Survey)	No
221	33220754	Demographic Correlates of Veterans' Adverse Social Determinants of Health	Cross-Sectional Study	No	N/A	At least one outpatient encounter or inpatient admission	Not specified	N/A	Association between demographics and adverse SDOH	Standard (ICD-10, VA specific codes)	No
225	35676746	Return Visits to the Emergency Department: An Analysis Using Group Based Curve Models	Retrospective Observational Study	No	N/A	Return visits to ED	Not specified	N/A	Association between return visits to ED and patient characteristics (clinical diagnosis, EHR, SDOH)	Standard (US Census data, data from Indiana Network for Patient Care)	
229	32739860	Social Determinants and Emergency Department Utilization: Findings from the Veterans Health Administration	Cross-Sectional Study	No	N/A	ED visits	Not specified	N/A	Association between ED visits and seven indicators of SDOH	Standard (ICD-10, VA specific codes)	No

231	34969350	Social Determinants Associated with Loss of an Eye in the United States Using the All of Us Nationwide Database	Case-controlled study	No	N/A	Loss of an eye	ocular trauma, ocular tumors, and severe endophthalmitis	N/A	Association between the loss of an eye and SDOH	Standard (ICD0-10, US Census data)	
234	35352489	Associations between Cardiometabolic Disease Severity, Social Determinants of Health (Sdoh), and Poor Covid-19 Outcomes	Retrospective Longitudinal Study	No	N/A	COVID-19 outcomes (hospitalization, ICU admission, mortality)	Cardiometabolic Diseases	Not specified	Outcome Measures (hospitalization, ICU admission, mortality), CMDS (Cardiometabolic Disease Staging)	Standard (CMDS scores, SDoH measures)	Yes
236	36381875	Socioeconomic and Comorbid Factors Affecting Mortality and Length of Stay in Covid-19 Patients	Retrospective Study	No	N/A	COVID-19 outcomes (Length of Stay and In-hospital Mortality)	COVID-19	Not specified	Outcome Measures (Length of Stay, In-hospital Mortality), Comorbidities (Elixhauser Comorbidity Index)	Standard (Elixhauser Comorbidity Index)	Yes
239	32880954	Relative Accuracy of Social and Medical Determinants of Suicide in Electronic Health Records	Observational Study	No	N/A	Suicide or intentional self-injury	Suicide and Self-Harm	Not specified	Predictive accuracy of suicide or intentional self-injury using ICD codes	Custom (Using ICD-9 codes for SDoH and medical diagnoses)	Yes
244	36161141	Food Insecurity, Social Needs, and Smoking Status among Patients in a County Hospital System	Retrospective Observational Study	No	N/A	Smoking	Smoking	Not specified	Association between smoking and food insecurity or other social needs	Custom (Hunger Vital Sign, National Association of Community Health Centers' Protocol for Responding to and Assessing Patients' Assets, Risks, and Experiences)	No
257	32675271	The Impact of Social and Clinical Complexity on Diabetes Control Measures	Retrospective cohort study	No	-	Poor glucose control (HbA1c > 9%)	Diabetes Mellitus	Not specifically mentioned	Quality of diabetes control (measured by HbA1c values), Charlson Comorbidity Index, Social Deprivation Index	Standard (utilized the Charlson Comorbidity Index and Social Deprivation Index)	No
260	31003812	The Impact of Social Determinants of Health on Hospitalization in the Veterans Health Administration	Retrospective Observational Study	No	N/A	Hospitalization (overnight stay in a VHA hospital)	Not specified	N/A	Association between hospitalization rates and SDOH variables	Standard (US Census data and American Community Survey)	No
266	36245804	Patient Characteristics and Neighborhood Attributes Associated with Hepatitis C Screening and Positivity in Philadelphia	Retrospective Observational Study	No	N/A	Hepatitis C Screening and Positivity	Hepatitis C	N/A	Association between hepatits screening/positivity and SDOH	Standard (US Census tract)	
267	36490368	Characterizing Primary Care Patients with Posttraumatic Stress Disorder Using Electronic Medical Records: A Retrospective Cross-Sectional Study	Retrospective cross-sectional study	No	-	PTSD diagnosis	PTSD	Not specified	IV: depreviation (e.g., material and social); DV: record of PTSD diagnosis ICD-9 code (309.81) or dianosis text with PTSD-related words	Standard and custom - IV: material deprivation (the lack of the goods and conveniences within the individual's dissemination area), social deprivation (social network including living alone, marital status, single-parent families); DV: PTSD = a constellation of specific disruptive psychiatric symptoms after having experienced, or witnessed, a traumatic event;	Yes

268	34212757	Area Deprivation Index and Cardiac Readmissions: Evaluating Risk-Prediction in an Electronic Health Record	Retrospective observational analysis	No	-	30-day and 1-year readmission and mortality following hospitalization	Heart Failure, Myocardial Ischemia, Atrial Fibrillation	Not specifically mentioned	Readmission and mortality rates, Cox proportional hazards models, C-statistic, net reclassification index	Standard (utilized Area Deprivation Index and other standard clinical variables)	Yes
269	28161034	Impact of Social Determinants of Health on Medical Conditions among Transgender Veterans	Retrospective observational analysis	No	-	Prevalence and associations of social determinants with medical conditions	Mood Disorder, PTSD, Alcohol Abuse Disorder, Illicit Drug Abuse Disorder, Tobacco Use Disorder, Suicidal Risk, HIV, Hepatitis C	Not specified	Prevalence of social determinants and their associations with medical conditions	Standard (utilized ICD-9 diagnosis codes and VA administrative data)	No
273	31831290	Association between Social and Economic Needs with Future Healthcare Utilization	Retrospective case/control analysis	No	-	Healthcare utilization (Primary care, emergency department, or urgent care visit at Kaiser Permanente Northwest from October 1, 2016 to November 31, 2017)	Not specified	Not specified	IV: Social or economic needs, DV: Future Hospitalization and ED visits	Standard and custom - IV: Social and economical needs (based on ICD-10-CM codes (e.g., insufficient social insurance or welfare support, homelessness, unemployment)); DV: Future hospital and ED visit (presence or absence of any hospitalization or ED visit in the 12 months)	Yes
281	28341220	National Academy of Medicine Social and Behavioral Measures: Associations with Self-Reported Health	Survey study	No	-	Associations of social and behavioral determinants of health with self-reported physical and mental health	Physical and Mental Health	Not specified	Associations between social and behavioral determinants and global physical and mental health	Standard (used NAM panel of SBD measures, SF-12 survey, MCSD Scale)	No
284	36112581	Associations of Four Indexes of Social Determinants of Health and Two Community Typologies with New Onset Type 2 Diabetes across a Diverse Geography in Pennsylvania	Nested Case-Control Study	No	N/A	Onset of Type 2 Diabetes	Type 2 Diabetes	N/A	Associations of SDOH indexes and community typologies with Type 2 Diabetes; Adjustments for confounding variables (age, sex, race, ethnicity, Medical Assistance status, and tobacco use)	Standard (Based on American Community Survey 2015 data for SDOH indicators)	No
285	36315199	Multiple Electronic Health Record-Based Measures of Social Determinants of Health to Predict Return to the Emergency Department Following Discharge	Retrospective cross-sectional observational study	No	-	Readmission	Not specified	Not specified	IV: Economic and social resources, Living and working conditions, personal health behaviors, health status and disability; DV: Return to the emergency department	Standard (Academic Health Science Center) and custom	Yes

286	35171031	Association of Unmet Social Needs with Chronic Illness: A Cross-Sectional Study	Observational Study	No	N/A	Common chronic illnesses (cancer, anemia, diabeter, etc.)	cancer (excluding skin cancers), anemia, diabetes, hyperlipidemia, substance use disorder, hypertension, psychiatric illness (including depression, anxiety, post-traumatic stress disorder), cardiovascular disease (including stroke, myocardial infarction, and congestive heart failure), obesity, chronic obstructive pulmonary disease, asthma, and chronic kidney disease	Not specified	Associations between social needs and chronic illnesses	Standard (ICD-10)	No
289	33041232	Association of Social Behaviors with Community Discharge in Patients with Total Hip and Knee Replacement	Retrospective cohort study	No	N/A	Community discharge after elective total joint arthroplasty	Joint arthroplasty	Not specified	Association between SDOH and community discharge after elective total joint arthroplasty	Standard (ICD-9 and ICD-10)	No
301	29893618	Race/Ethnicity, Socioeconomic Status, and Healthcare Intensity at the End of Life	Retrospective Observational Study	No	N/A	End of life (Death)	Cancers with poor prognoses, chronic pulmonary disease, coronary artery disease, heart failure, severe chronic liver disease, chronic renal failure, dementia, diabetes with end-organ damage, and peripheral vascular disease.	N/A	Association between race/ethnicity, social determinants of health, and healthcare intensity in the last 30 days of life for individuals with chronic, life-limiting illness	Standard (ICD-10)	No
302	35704625	Associations between Healthcare Utilization and Access and Diabetic Retinopathy Complications Using All of Us Nationwide Survey Data	Retrospective cohort study	No	N/A	Healthcare utilization and access	Diabetic retinopathy	N/A	Associations between healthcare utilization and access and diabetic retinopathy complications	Standard (ICD-10)	No
304	34848998	Social Determinants of Health Correlating with Mechanical Ventilation of Covid-19 Patients: A Multi-Center Observational Study	Multi-Center Observational Study	Yes	Race, Poverty, Disability	Mechanical Ventilation of COVID-19 Patients	COVID-19	Not Specified	association between SDOH and mechanical ventilation (MV).	Standard (EMR + US Census)	Yes
308	35688597	Multilevel Determinants of Racial/Ethnic Disparities in Severe Maternal Morbidity and Mortality in the Context of the Covid-19 Pandemic in the USA: Protocol for a Concurrent Triangulation, Mixed-Methods Study	Retrospective Study	Yes	racial/ethnic	severe maternal morbidity and mortality (SMMM) during covid-19	SMMM	Not Specified	racial/ethnic disparities in SMMM with COVID-19	Standard (Data from S3C, N3C, ACS, AHA combined)	No
324	24886134	Community-Level Determinants of Obesity: Harnessing the Power of Electronic Health Records for Retrospective Data Analysis	Retrospective observational study	No	N/A	Obesity	Obesity	N/A	Association between obesity and SDOH	Custom (OSUWMC, Nielsen PrimeLocation, NAICS, Manual compilation of directories)	No
333	28708848	Predicting All-Cause Risk of 30-Day Hospital Readmission Using Artificial Neural Networks	Retrospective observational study	No	N/A	30-day readmission	Not specified	N/A	Association between 30-day readmission and patient EHR/SDOH data	Standard (American Community Survey ,US Census data	No

335	32967923	Prevalence of Social Risks on Inpatient Screening and Their Impact on Pediatric Care Use	Survey study	No	N/A	Pediatric Care/Not Specified	Pediatric Care/Not Specified	N/A	Multiple, including LOS Increase and complex chronic conditions	Custom (screening survey was developed in 2015 by a multidisciplinary group)	No
344	28438048	The Impact of Infant Well-Child Care Compliance and Social Risks on Emergency Department Utilization	Retrospective Observational Study	No	N/A	ED (Emergency department) visits	ED visits (No specific diseases mentioned)	N/A	Associations between the number of completed well-child visits or reported social risks and ED utilization	Standard (Emergency Severity Index)	No
346	36503669	Caries Risk and Social Determinants of Health: A Big Data Report	Retrospective cross-sectional study	No	N/A	Caries risk	Caries	N/A	Association between caries risk and SDOH (SDI)	Standard (CDT (Current Dental Terminology), Social Deprivation Index in ACS)	No
349	33215073	Using Electronic Health Records for Population Health Sciences: A Case Study to Evaluate the Associations between Changes in Left Ventricular Ejection Fraction and the Built Environment	Retrospective observational study	No	N/A	Changes in ejection fraction	Heart failure	N/A	Associations between SDOH and changes in ejection fraction (EF)	Standard (ICD-9, ICD-10)	No
350	35656210	Health Care Utilization among Women of Reproductive Age Living in Public Housing: Associations across Six Public Housing Sites in San Francisco	Retrospective Study	No	N/A	Outpatient and ED visits	N/A	Not Specified	incidence rate ratios (IRR) for outpatient visits (count) and odds ratios (OR) for ED visit	Standard	No
371	32860253	Relationship of Neighborhood Social Determinants of Health on Racial/Ethnic Mortality Disparities in Us Veterans-Mediation and Moderating Effects	Retrospective cohort study	No	N/A	Mortality (Death)	Not specified	N/A	Association between SDOH and mortality rates	Custom (Area deprivation index, also standard (US Census Data))	No
373	33079198	Patient-Reported Social and Behavioral Determinants of Health and Estimated Risk of Hospitalization in High-Risk Veterans Affairs Patients	Survey study	No	N/A	90-day and 180-day all-cause hospitalizations	Not specified	N/A	Estimation of 90-day and 180-day hospitalization risk using SDOH data	Custom (VA specific definitions)	Yes
378	35308954	Testing of a Risk-Standardized Complication Rate Electronic Clinical Quality Measure (Ecqm) for Total Hip and/or Total Knee Arthroplasty	Observational retrospective cohort study	No	N/A	Complications within 90 days of Total Hip and/or Total Knee Arthroplasty	Total Hip and/or Total Knee Arthroplasty	N/A	Assessment of the risk-standardized complication rate (RSCR) following elective primary total hip (THA) and knee arthroplasty (TKA) at the clinician group level.	Custom (NQF1550 and NQF3493)	No
382	34348133	The Association between Social Needs and Chronic Conditions in a Large, Urban Primary Care Population	Observational Study	No	N/A	Associations of social determinants with chronic medical conditions	Smoking, alcohol/drug use disorder, obesity, hypertension, diabetes, asthma, depression	N/A	Associations between social needs and specific chronic conditions using social needs screening and clinical data from Electronic Health Records	Standard (ICD-10)	No
383	33622817	Association of Covid-19 with Race and Socioeconomic Factors in Family Medicine	Retrospective Study	Yes	Race	Patients referred for SARS CoV-2 testing	COVID-19	N/A	Odds Ratio, Area Deprivation Index	Standard (EPIC + CRISP)	No
401	36279173	Medication Adherence and Cardiometabolic Control Indicators among American Indian Adults Receiving Tribal Health Services: Protocol for a Longitudinal Electronic Health Records Study	Observational longitudinal study	No	N/A	Cardiometabolic control in American-Indian adults with Type 2 Diabetes	Type 2 Diabetes	Medication adherence	bivariate relationship between medication aherence and cardiometabolic control indicators, patient demographics and comorbidities	Standard (ICD-10)	Yes

403	35484624	Prospective Evaluation of Social Risks, Physical Function, and Cognitive Function in Prediction of Non-Elective Rehospitalization and Post-Discharge Mortality	Prospective cohort study	No	N/A	Non-elective rehospitalization and post-discharge mortality	Not specified	N/A	Prediction of non-elective rehospitalization and post-discharge mortality using SDOH and other variables	Custom (Transitions Support Level)	Yes
407	31745856	Social Determinants and Military Veterans' Suicide Ideation and Attempt: A Cross-Sectional Analysis of Electronic Health Record Data	Retrospective cross-sectional study	No	N/A	Suicide Ideation and Attempt	Not specified	N/A	Association between suicide ideation/attempt and SDOH	Standard (ICD-10) and VA-specific (VHA's Suicide Prevention Applications)	No
408	33428455	Empiric Evidence of Ethnic Disparities in Coronavirus Positivity in Washington State	Observational cohort study	No	N/A	Coronavirus positivity and hospitalization	Coronavirus infections	N/A	Association between ethnicity and outcomes of coronavirus positivity and hospitalization	Standard (ICD-10, American Community Survey)	No
409	32851616	Toward Understanding the Value of Missing Social Determinants of Health Data in Care Transition Planning	Retrospective cross-sectional study	No	-	30-day revisit	Not specified	Not specified	IV: Activities of daily living (ADL), Abuse, Alcohol, Employment/School, Exercise, Home environment, Nutrition, Sexual, Substance abuse, Tobacco; CV: deprivation index, DV: 30-day revisit	Not specified, assuming standard (the Cerner EHR at the University of Alabama at Birmingham Medical Center (UABMC)	Yes
410	32150271	Association of Neighborhood Measures of Social Determinants of Health with Breast, Cervical, and Colorectal Cancer Screening Rates in the Us Midwest	Retrospective cross-sectional study	No	N/A	Breast, Cervical, and Colorectal Cancer Screening Rates	Breast, Cervical, and Colorectal Cancer	N/A	Association between SDOH and screening for breast, cervical, and colorectal cancer	Standard (ICD-10, US Census)	No
416	34948748	Differences in Covid-19 Risk by Race and County-Level Social Determinants of Health among Veterans	Retrospective Study	Yes	Race, income /housing	Covid-19 Risk	Covid-19	N/A	association between county-level SDOH and COVID-19	Standard (VA EHR)	No
419	36166652	Understanding Racial Disparities in Covid-19-Related Complications: Protocol for a Mixed Methods Study	Mixed Methods (Retrospective + Survey)	Yes	Race	racial disparities in severe COVID-19	Covid-19	N/A	dramatic disparity in COVID-19 severity (ie, hospitalization, mortality)	Standard (Kaiser EMR)	No
424	34820853	Your Neighborhood Matters: A Machine-Learning Approach to the Geospatial and Social Determinants of Health in 9-1-1 Activated Chest Pain	Retrospective Observational Study	No	N/A	Calling 9–1-1 for acute chest pain	Acute Coronary Syndrome (ACS)	N/A	Associations between the geospatial and social determinants of health	Standard (American Heart Association definition for ACS)	No
426	32584879	Assessing the Impact of Social Determinants of Health on Predictive Models for Potentially Avoidable 30-Day Readmission or Death	Retrospective cohort study	No	N/A	Potentially avoidable 30-day readmission or death after a hospital admission	Not specified	N/A	Association between SDOH and potentially avoidable hospital readmission or death 30 days after a hospital admission	Custom (American Community Survey, Center for Disease Control and Prevention, United States Department of Agriculture, United States Environmental Protection Agency, the FACETS dataset, and New York City Open Data)	Yes

429	32598228	Assessing the Impact of Social Needs and Social Determinants of Health on Health Care Utilization: Using Patient- and Community-Level Data	Retrospective cross-sectional study	No	-	Healthcare utiilization	Not specified	Not specified	IV: Housing issues, Area Deprivation; DV: Healthcare utilization	Standard (Area Deprivation Index (from ACS 5-Year Estimates in 2017)) and Custom (Hospital utilization(the number of encounters with the health care syste); Housing issues (homelessness, housing instability, and structural challenges associated with patients' residence (eg, characteristics of a building such as existing lead paint or structural damage)))	Yes
430	35396996	Assessing Socioeconomic Bias in Machine Learning Algorithms in Health Care: A Case Study of the Houses Index	Cross-Sectional Study	No	N/A	Algorithmic bias for pediatric asthmatics	Asthma	N/A	Association between data quality of electronic health records (EHRs) and low socioeconomic status (SES)	Standard (Predetermined Asthma Criteria (PAC))	Yes
439	35570956	Analyzing Relationships between Economic and Neighborhood-Related Social Determinants of Health and Intensive Care Unit Length of Stay for Critically Ill Children with Medical Complexity Presenting with Severe Sepsis	Retrospective Observational Study	No	N/A	Admission to the pediatric critical care complex with severe sepsis	Severe sepsis	N/A	Association between PICU (Pediatric Intensive Care Unit) length of stay and socioeconomic and neighborhood related factors	A severe sepsis algorithm (29) incorporated into an existing electronic medical record (EMR), Cerner® Powerchart	No
447	29492953	Food Insecurity in Older Adults in an Integrated Health Care System	Retrospective cohort study	Possibly?	Examined bias in survey completion	Not specified (members of Kaiser Permanente Colorado)	Not specified	N/A	IV: Sociodemographic, Health Status, Quality of Life, Functional Abilities, and Social Needs; DV: Food insecurity	Standard (all IV based on EHR) and custom (food security)	Yes
449	34401723	Neighborhood Socioeconomic Status, Health Insurance, and Ckd Prevalence: Findings from a Large Health Care System	Retrospective observational study	No	N/A	CKD prevalence	Chronic Kidney Disease	N/A	Association between SDOH / individual-level health insurance status and CKD prevalence	Standard (American Community Survey)	No
458	33168270	Role of Health Insurance and Neighborhood-Level Social Deprivation on Hypertension Control Following the Affordable Care Act Health Insurance Opportunities	Longitudinal observational study	No	N/A	Affordable Care Act (ACA) Medicaid expansion	Hypertension	Affordable Care Act (ACA) Medicaid expansion	gaining health insurance, neighborhood-level social deprivation, hypertension control	Standard (social deprivation: Social Deprivation Index (SDI) by American Community Survey; hypertension: blood pressure) and custom (newly insured/continuously insured/discontinuosly uninsured/continuosly uninsured)	Yes

463	31445257	Prediction of Emergency Department Revisits Using Area-Level Social Determinants of Health Measures and Health Information Exchange Information	Retrospective cross-sectional study	No	N/A	Emergency department revisits	Not specified	N/A	IV: characteristic, health record, SDoH (including socioeconomic, material, substance-related behaviors, environmental, health service, social contexts)	Standard (EHR and Census) and Custom (Emergency department revisits: revisit only within 30days)	Yes
469	33006568	Investigation of Cardiovascular Health and Risk Factors among the Diverse and Contemporary Population in London (the Together Study): Protocol for Linking Longitudinal Medical Records	cross-sectional observational study unfolding into prospective longitudinal study	No	N/A	Cardiovascular health and risk factors	Cardiovascular diseases	N/A	cardiovascular disease incidence and mortality	Standard (NHS)	Yes
470	35612181	Covid-19 Positivity Differences among Patients of a Rural, Southern Us State Hospital System Based on Population Density, Rural-Urban Classification, and Area Deprivation Index	Retrospective Study	Yes	Rural vs Urban, Area Deprivation Index	Covid-19 Positivity	Covid-19	N/A	SDOH and rural/urbam classification and area deprivation index (ADI)	Standard	No
476	29421603	Associations of Neighborhood-Level Racial Residential Segregation with Adverse Pregnancy Outcomes	Retrospective observational study	No	N/A	The rate of adverse pregnancy outcomes	Adverse pregnancy outcomes (preterm birth, spontaneous preterm birth, medically indicated preterm birth, and small for gestational age)	N/A	Associations between neighborhood-level racial residential segregation with adverse pregnancy outcomes	Custom (US Census tracts and Getis-Ord Gi* statistic to measure segregation)	No
479	31134134	A Social Determinant of Health May Modify Genetic Associations for Blood Pressure: Evidence from a Snp by Education Interaction in an African American Population	Retrospective observational study	No	N/A	The rate of Systolic Blood Presssure (SBP) and Diastolic Blood Pressure (DBP)	Systolic Blood Presssure (SBP) and Diastolic Blood Pressure (DBP)	N/A	Association between SDOH (education level) and a common genetic variant with blood pressure in an African American population	Standard (ICD-9 CM)	No
481	30003015	Association of Behavioral Health Factors and Social Determinants of Health with High and Persistently High Healthcare Costs	Prospective observational study	No	N/A	Diagnosis of psychiatric disorders	Psychiatric disorders	N/A	IV: health care costs, clinical diagnoses, patient activation and engagement, SDoH (including SES, living situation, legal and conservatorship status; access to mobile or electronic healthcare, neighborhood environment, transportation access); ACEs, IPV, Patient Activation; DV: Psychiatric and behavioral disorder (e.g., depression, anxiety, substance use)	Standard (e.g., psychiatric and behavioral disorders: EHR-based ICD-9 diagnoses; Adverse Childhood Experiences score)	Yes
483	32727486	A Model of Disparities: Risk Factors Associated with Covid-19 Infection	Retrospective Study	No	N/A	risk of COVID-19 infection	Covid-19	N/A	Odds ratio for all risk factors1	Standard	Yes
485	30010764	Racial Residential Segregation and Hypertensive Disorder of Pregnancy among Women in Chicago: Analysis of Electronic Health Record Data	Retrospective observational study	No	N/A	The rate of hypertensive disorder of pregnancy (HDP)	Hypertensive disorder of pregnancy (HDP)	N/A	Association between racial segregation and HDP	Custom (US Census tracts and Getis-Ord Gi* statistic to measure segregation)	No
486	29382633	Prediction of Incident Hypertension within the Next Year: Prospective Study Using Statewide Electronic Health Records and Machine Learning	Retrospective and prospective observational study	No	N/A	Risk prediction of hypertension	Incident essential hypertension	N/A	1-year risk prediction model for incident essential hypertension	Standard (ICD-9)	Yes

487	30646172	Value of Neighborhood Socioeconomic Status in Predicting Risk of Outcomes in Studies That Use Electronic Health Record Data	Cohort study	No	N/A	Use of health care services (emergency department and inpatient and outpatient encounters) and hospitalizations	Accidents, asthma, influenza,myocardial infarction, and stroke	N/A	IV: clinical data (demographics, comorbidities, laboratory tests, medications, and use of health care services), Neighborhood Socioeconomic Status; DV: Utilization of healthcare	Standard (Neighborhood socioeconomic status: Agency for Healthcare Research and Quality (AHRQ) SES index) and custom (Utilization of healthcare: use of services including emergency department, inpatient and outpatient encounters, hospitalizations)	Yes
491	29574461	Police-Recorded Crime and Disparities in Obesity and Blood Pressure Status in Chicago	Retrospective cross-sectional study	No	N/A	The rate of obesity and elevated blook pressure	Obesity and elevated blood pressure (BP)	N/A	Associations between several types of police-recorded crime (violent, nonviolent, and homicide) and cardiometabolic health (obesity and elevated blood pressure [BP])	Custom (City of Chicago Police Data Portal)	No
492	30763292	Clinical and Sociobehavioral Prediction Model of 30-Day Hospital Readmissions among People with Hiv and Substance Use Disorder: Beyond Electronic Health Record Data	Randomized controlled trial	No	N/A	30-day readmission	HIV-positive substance users	financial incentives	IV: demographic; socio-economic variables (i.e., income, housing, employment, education, prior incarceration, violence social support), substance use, health record; DV: 30-day readmission	Standard (i.e., Addiction Severity Index, HIV dementia scale..)	Yes
493	36935292	The Importance of Social Risk Factors for Patients Diagnosed with Opioid Use Disorder	Retrospective cohort study	No	N/A	Diagnosed Opioid use disorder AND prescribed relavant medication	Opioid Use Disorder	N/A	IV: demographic, health record, SDoH (i.e., Food insecurity, housing, social connection, transportation)	Standard (demographic, health record, objective neighborhood information) and Custom (subjective social environment infomration)	Yes
495	31202286	Influence of Social Determinants of Health and Substance Use Characteristics on Persons Who Use Drugs Pursuit of Care for Hepatitis C Virus Infection	Retrospective observational study	No	N/A	The likelihood to pursue HCV management	Substance use, hepatitis C virus, opioid use disorder (OUD)	N/A	Associations between SDOH and linkage-to-care for HCV	Custom (New York State Office of Alcohol and Substance Abuse Services questionnaire)	No
496	34496419	Telemedicine Adoption During the Covid-19 Pandemic: Gaps and Inequalities	Retrospective Study	No	N/A	usage of telemedicine services	Not Specified	N/A	correlation of SDOH with telemedicine services	Custom(EHR + Cemsus Data)	Yes (Linear Regression)
509	36901047	Impact of Contextual-Level Social Determinants of Health on Newer Antidiabetic Drug Adoption in Patients with Type 2 Diabetes	Retrospective cohort study	No	N/A	Antidiabetic Drug Adoption in Patients with Type 2 Diabetes	Type 2 Diabetes	N/A	Association between SDOH and newer antidiabetic drug adoption	Custom (USDA Food Access Research Atlas, National Walkability Index etc.)	No
510	34328797	Racial Differences in Patient Portal Activation and Research Enrollment among Patients with Prostate Cancer	Observational Study	No	N/A	Patient portal activation and research participation among patients with prostate cancer	Prostate cancer	N/A	Association between patient portal activation-research enrollment and social determinants	Standard (ICD-10)	No

511	32878515	Change of Address as a Measure of Housing Insecurity Predicting Rural Emergency Department Revisits after Asthma Exacerbation	Restrospective obserational study	No	N/A	Emergency department revisits following asthma exacerbation	Asthma	Not specified	Multivariate logistic regression with odds ratios and confidence intervals for association between address change and ED revisits	Standard	No
513	33022319	Adverse Social Factors and All-Cause Mortality among Male and Female Patients Receiving Care in the Veterans Health Administration	Retrospective observational study	No	N/A	Mortality (death)	Not specified	N/A	Association between SDOH and all-cause mortality in the Veterans Health Administration	Custom (ICD-10 and VHA-specific classifications such as housing instability or military sexual trauma)	No
517	36208711	Race and Socioeconomic Status Are Associated with Inferior Patient-Reported Outcome Measures Following Rotator Cuff Repair	Retrospective cohort study	No	N/A	Patient-reported outcome measures following rotator cuff repair	Rotator Cuff Repair	N/A	Association between inferior patient-reported outcome measures following rotator cuff repair	Standard (PROMIS, US Census)	No
518	34261446	Evaluating the Association of Social Needs Assessment Data with Cardiometabolic Health Status in a Federally Qualified Community Health Center Patient Population	Observational cross-sectional study	No	N/A	Cardiometabolic health status	cardiometabolic diseases cuh as obesity, hypertension, atherosclerotic cardiovascular disease	Not specified	Stepwise and LASSO regression examining relationship between social needs screening responses and cardiometabolic health	Custom (EHR and Protocol for Responding to and Assesing Patients' Assets, Risks and Experiences)	
521	36630557	Impacts of Individual Patient Language and Neighborhood Ethnic Enclave on Covid-19 Test Positivity among Hispanic/Latinx Patients in San Francisco	Retrospective Study	No	N/A	COVID-19 positivity	Covid-19	Not specified	odds ratio (patients living in the highest versus lowest Hispanic/Latinx enclave)	Standard	No
526	36931038	Erasing Inequality: Examining Discrepancies between Electronic Health Records and Patient Narratives to Uncover Perceived Stigma and Dismissal in Clinical Encounters	Qualitative and semi-structural interview study	No	N/A	Not specified	Not specified	Not specified	Perceived stigma, its interactions with the health system, patients' feeling toward conflict between clinicians and patients understanding	Not specified	No
532	26944212	Differences in Access to and Preferences for Using Patient Portals and Other Ehealth Technologies Based on Race, Ethnicity, and Age: A Database and Survey Study of Seniors in a Large Health Plan	Retrospective Study	No	N/A	N/A	N/A	Not specified	No	N/A	No
533	31862438	Neighborhood Disadvantage and Lung Cancer Incidence in Ever-Smokers at a Safety Net Health-Care System: A Retrospective Study	Retrospective Study	No	N/A	lung cancer incidence in ever-smokers	lung cancer	Not specified	multivariate Cox-proportional hazard model	Standard	No
538	34053276	Social Determinants of Health in Total Knee Arthroplasty : Are Social Factors Associated with Increased 30-Day Post-Discharge Cost of Care and Length of Stay?	Retrospective Study	No	N/A	the need for total knee arthroplasty30-day length of stay (LOS) and total cost of care (TCOC) after total knee arthroplasty (TKA)	total knee arthroplasty	Not specified	multiple regression analyses	standard	No
548	28093689	Characterizing a "Big Data" Cohort of over 200,000 Low-Income U.S. Infants and Children for Obesity Research: The Advance Early Life Cohort	Retrospective Study	No	N/A	early life obesity	obesity	Not specified	descriptive analysis focusing on the availability of BMI measures, sociodemographic characteristics, weight status, and longitudinal follow-up of the cohort	Standard	No

549	37070611	Improving Outcomes of Pediatric Lupus Care Delivery with Provider Goal Setting Activities and Multidisciplinary Care Models	Retrospective Study	No	N/A	care quality and its association with prednisone use in youth with systemic lupus erythematosus (SLE)	Systemic Lupus Erythematosus (SLE)	Not specified	Pediatric Lupus Care Index (pLCI) performance, associations between pLCI and subsequent prednisone use	Custom (pediatric Lupus Care Index (pLCI))	No
550	31704758	The Association between Neighborhood Socioeconomic and Housing Characteristics with Hospitalization: Results of a National Study of Veterans	Observational cohort study	No	N/A	Hospitalization rates	Not speificed	Not specified	generalized estimating equation	Custom (defined hospitalization based on specific criteria within the VHA system)	No
558	33147232	Racial-Ethnic Differences in Prevalence of Social Determinants of Health and Social Risks among Middle-Aged and Older Adults in a Northern California Health Plan	Retrospective Study	No	N/A	N/A	N/A	Not specified	age-sex standardized estimates, multivariable logistic regression	Custom	No
562	36739248	The Bari-Hoods Project: Neighborhood Social Determinants of Health and Postoperative Weight Loss Using Integrated Electronic Health Record, Census, and County Data	Observational study	No	N/A	Weight loss following bariatric surgery.	Obesity	Not specified	percent total weight lost (%TWL)	Custom (Integration of EHR, census, and county data for specific variables related to weight loss following bariatric surgery)	No
564	35940570	Geographic Variation in Acute Pediatric Mental Health Utilization	retrospective study	No	N/A	Admission for a mental health diagnosis or symptoms	Mental health issues in pediatric patients	Not specified	Normalized visit rates	Custom (EHR and Community survey data)	No
565	29042345	The Association of Patient Factors, Digital Access, and Online Behavior on Sustained Patient Portal Use: A Prospective Cohort of Enrolled Users	Prospective cohort study	No	N/A	N/A	N/A	Not specified	the frequency of portal log-ins	Custom	No
571	34009238	Association of Socioeconomic Disadvantage with Long-Term Mortality after Myocardial Infarction: The Mass General Brigham Young-Mi Registry	Retrospective cohort study	No	N/A	Myocardial infarction (MI) experienced by patients at or before 50 years of age	Myocardial Infarction (MI)	Not specified	all-cause and cardiovascular mortality	Custom	No
572	31367515	Identifying Neighborhood Characteristics Associated with Diabetes and Hypertension Control in an Urban African-American Population Using Geo-Linked Electronic Health Records	Retrospective cohort study	No	N/A	diabetes and hypertension control	diabetes and hypertension	Not specified	poor diabetes control (defined as hemoglobin A1c > 9% or no record of a HbA1c test in the previous year) and poor hypertension control (defined as systolic blood pressure ≥ 140 mmHg or diastolic blood pressure ≥ 90 mmHg).	standard(Health Resources and Services Administration (HRSA) Uniform Data System (UDS) reporting)	No
577	31414132	Association of Rising Violent Crime with Blood Pressure and Cardiovascular Risk: Longitudinal Evidence from Chicago, 2014-2016	Retrospective cohort study	No	N/A	association between rising violent crime and elevated blood pressure (BP)	hypertension	Not specified	elevated blood pressure (BP)	Standard	Yes
579	29143960	Predicting Frequent Emergency Department Use among Children with Epilepsy: A Retrospective Cohort Study Using Electronic Health Data from 2 Centers	Retrospective cohort study	No	N/A	use of the emergency department (ED) among children with epilepsy	epilepsy	Not specified	frequency of emergency department usage.	Standard	Yes
587	27742630	Predicting Frequent Hospital Admission Risk in Singapore: A Retrospective Cohort Study to Investigate the Impact of Comorbidities, Acute Illness Burden and Social Determinants of Health	Observational, retrospective study	No	N/A	frequent hospital admissions	Not specified	Not specified	frequent hospital admissions, evaluated using the Area Under the Receiver Operating Characteristic Curve (AUC)	Standard	Yes

592	32160902	Epigenetic Predictors of All-Cause Mortality Are Associated with Objective Measures of Neighborhood Disadvantage in an Urban Population	prospective cohort study	No	N/A	association between neighborhood characteristics and epigenetic biomarkers of mortality risk	Not specified	Not specified	epigenetic mortality risk score (eMRS)	Standard	No
601	36877520	Association of Education with Dementia Incidence Stratified by Ethnicity and Nativity in a Cohort of Older Asian American Individuals	Retrospective study	Yes	Not specified	incident dementia diagnosis in the EHR	dementia	Not specified	dementia incidence rates	Standard	No
610	34348475	Rheumatic Heart Disease in the United States: Forgotten but Not Gone: Results of a 10 Year Multicenter Review	retrospective study	No	N/A	children living with acute rheumatic fever (ARF) and rheumatic heart disease (RHD)	Acute Rheumatic Fever (ARF) and Rheumatic Heart Disease (RHD)	Not specified	regression analysis to investigate the relationships between community deprivation and outcomes	Standard	No
613	36964218	Obesity in Pediatric Congenital Heart Disease: The Role of Age, Complexity, and Sociodemographics	retrospective cohort study	No	N/A	children with and without congenital heart disease (CHD)	congenital heart disease (CHD) and obesity	Not specified	logistic regression analysis to compare the odds of obesity between children with CHD and a control population of non-CHD patients	standard	No
620	30030110	Neighbourhood Socioeconomic Disadvantage, Risk Factors, and Diabetes from Childhood to Middle Age in the Young Finns Study: A Cohort Study	retrospective cohort study	No	N/A	diabetes and cardiometabolic risk factors	obesity, hypertension, fatty liver, and diabetes	Not specified	Logistic and linear regression analyses were used to assess factors	standard	No
636	36375010	Parental Childhood Adversity and Pediatric Emergency Department Utilization: A Pilot Study	cross-sectional survey	No	N/A	pediatric emergency department (PED) utilization	Not specified	Not specified	Pearson correlation to analyze the relationship between parental adverse childhood experiences (ACEs), resilience, social determinants of health, and PED utilization	standard	No
639	36038962	Neighborhood Deprivation and Severe Maternal Morbidity in a Medicaid-Insured Population in Georgia	retrospective study	No	N/A	severe maternal morbidity (SMM) among individuals in a public health system in Atlanta, Georgia	severe maternal morbidity (SMM)	Not specified	log-binomial regression with generalized estimating equations to estimate relative risks (aRR) for the association between neighborhood deprivation and SMM	standard	No
640	29569327	Reducing Emergency Room Visits and in-Hospitalizations by Implementing Best Practice for Transitional Care Using Innovative Technology and Big Data	retrospective study	No	N/A	reducing unnecessary hospitalizations in a Medicaid population by identifying and acting upon social determinants of health	Not specified	Not specified	use negative binomial regression to compare utilization before and during the intervention	Standard	No
648	36958532	Social Needs as a Risk Factor for Positive Postpartum Depression Screens in Pediatric Primary Care	retrospective cohort study	No	N/A	the association between self-reported social needs and postpartum depression (PPD) symptoms in mothers	postpartum depression (PPD)	Not specified	logistic regression	Standard	No
649	36287625	Predictors of Completion of Clinically Necessary Radiologist-Recommended Follow-up Imaging: Assessment Using an Automated Closed-Loop Communication and Tracking Tool	retrospective study	No	N/A	the completion of clinically necessary follow-up imaging in patients with adverse social determinants of health	Not specified	Not specified	Multivariable logistic regression analysis	Standard	No

651	34709424	Exercise Facilities and the Prevalence of Obesity and Type 2 Diabetes in the City of Madrid	retrospective cohort study	No	N/A	the association between the availability of exercise facilities and the likelihood of obesity and type 2 diabetes in the adult	obesity and type 2 diabetes	Not specified	Poisson regression models with robust standard errors clustered	standard	No
653	36683912	Associating Social Determinants of Health with Promis Cat Scores and Health Care Utilization after Acl Reconstruction	retrospective cohort study	No	N/A	the outcomes of anterior cruciate ligament reconstruction (ACLR) surgery	anterior cruciate ligament reconstruction	Not specified	the PROMIS computer adaptive test (CAT) scores	standard	No
655	35184126	Socioeconomic Correlates of Keratoconus Severity and Progression	retrospective cohort study	No	N/A	keratoconus	keratoconus	Not specified	employed logistic regression to evaluate factors associated with severity at presentation and corneal transplantation, and Cox proportional hazards modeling to evaluate disease progression	standard	No
657	35813187	Prevalence of Social and Economic Stressors among Transgender Veterans with Alcohol and Other Drug Use Disorders	retrospective study	Yes	social and econo mic stressor s	the prevalence of social and economic stressors among transgender and cisgender VA outpatients, particularly in the context of alcohol and drug use disorders (AUD and DUD)	Alcohol Use Disorder (AUD) and Drug Use Disorder (DUD)	Not specified	Minority Stress Model	standard	No
659	35455496	Family Stressors and Resources as Social Determinants of Health among Caregivers and Young Children	cross-sectional study	No	N/A	various health outcomes related to caregiver and child health, specifically caregiver depressive symptoms, anxiety symptoms, and sleep problems	Not specified	Not specified	Cumulative Stressor Inventory and Cumulative Resources Inventory	standard and custom	No
661	32367203	Geospatial Variations and Neighborhood Deprivation in Drug-Related Admissions and Overdoses	retrospective cohort study	No	N/A	Spatial heterogeneity in patients admitted for opioid, amphetamine, and psychostimulant-related diagnoses and overdoses	drug use, specifically opioid, amphetamine, and psychostimulant use and overdoses	Not specified	geospatial clustering of substance use-related admissions and overdoses	standard	No
665	36389291	The Impact of Substance Use on Health Care Utilization, Treatment, and Outcomes in Patients with Non-Small Cell Lung Cancer		No	N/A	the impact of substance use on health care utilization, treatment, and outcomes in patients with non-small cell lung cancer (NSCLC)	non-small cell lung cancer (NSCLC)	Not specified	comparative and multivariable analyses to evaluate lung cancer outcomes including survival	standard	No
669	35947491	Telehealth to Improve Continuity for Patients Receiving Buprenorphine Treatment for Opioid Use Disorder	retrospective review	No	N/A	continuity of care for patients receiving buprenorphine treatment for opioid use disorder	opioid use disorder	Not specified	rates of continuity by visit type (in-person or telehealth)	standard	No

674	34529635	Longitudinal Trends in Body Mass Index before and During the Covid-19 Pandemic among Persons Aged 2-19 Years - United States, 2018-2020	observational study	Yes	selection bias and potential biases	the increase in body mass index (BMI) and the associated rise in obesity rates among children and adolescents during the COVID-19 pandemic	obesity	Not specified	the rate of BMI increase, obesity prevalence, and changes in these metrics before and during the COVID-19 pandemic	standard	No
678	35493405	Social Determinants of Human Papillomavirus Vaccine Series Completion among U.S. Adolescents: A Mixed-Methods Study	retrospective study	No	N/A	the completion of the human papillomavirus (HPV) vaccine series among U.S. adolescents	HPV-associated cancers	Not specified	multivariable logistic regression analyses to examine the influence of various factors on timely series completion, with significance set at 0.05. Adjusted odds ratios (aORs) and 95% confidence intervals (CIs) were reported to address effect size	standard	No
681	33519165	The Influence of Health Insurance Stability on Racial/Ethnic Differences in Diabetes Control and Management	retrospective study	No	N/A	the management and control of type 2 diabetes mellitus	type 2 diabetes mellitus	Not specified	diabetes management measures established by the National Committee for Quality Assurance (NCQA)	standard	No
682	35211396	Social Disparities of Pain and Pain Intensity among Women Diagnosed with Early Stage Breast Cancer	retrospective study	No	N/A	the occurrence and intensity of pain in women diagnosed with early-stage breast cancer	breast cancer	Not specified	zero-inflated Poisson models to analyze pain occurrence and intensity	standard	No
696	32716125	Household Material Hardships Impact Emergency Department Reliance in Pediatric Patients with Sickle Cell Disease	retrospective study	No	N/A	Emergency department (ED) utilization in pediatric patients with sickle cell disease (SCD)	Sickle cell disease (SCD) in pediatric patients	identify modifiable risk factors (household material hardships) for high ED utilization to potentially guide interventions	emergency department reliance (EDr); P-values and R-squared (R2) values	Standard	No
698	35342390	The Impact of Aces on Bmi: An Investigation of the Genotype-Environment Effects of Bmi	retrospective study	No	N/A	the impact of Adverse Childhood Experiences (ACEs) on adult obesity	obesity	Not specified	linear regression, the association between adverse childhood experiences (ACEs) and body mass index (BMI)	Standard	No
699	31811046	A Statistical-Learning Model for Unplanned 7-Day Readmission in Pediatrics	retrospective cohort study	No	N/A	pediatric 7-day unplanned readmissions	Not specified	Not specified	LASSO regression assesse multicollinearity based on the area under the curve (AUC)	standard	Yes
700	37104833	Food Insecurity, Neighborhood, and Health Care Utilization in Health System Adults	Retrospectivl study	No	N/A	acute health care utilization within 90 days of hospital discharge	Not specified	Not specified	Multivariable negative binomial regression	Standard	No

701	33004699	Diabetes Prevalence and Demographics in 25 First Nations Communities in Northwest Ontario (2014-2017)	retrospective diabetes prevalence study	No	N/A	epidemiology and prevalence of diabetes in the First Nations communities in northwest Ontario, along with cardiovascular comorbidities in the adult population	Diabetes, along with comorbid hypertension and dyslipidemia	Not specified	Diabetes prevalence calculation	Standard	No
703	35306931	Racial Disparities in Covid-19 Test Positivity among People Living with Hiv in the United States	retrospective study	No	N/A	COVID-19 positivity in relation to HIV status and race/ethnicity	COVID-19 and HIV	Not specified	Multivariable logistic regression (odds ratios (aOR) and confidence intervals (CI))	standard	No
711	36265163	Machine Learning Techniques to Explore Clinical Presentations of Covid-19 Severity and to Test the Association with Unhealthy Opioid Use: Retrospective Cross-Sectional Cohort Study	Retrospective cohort study	No	N/A	COVID-19 severity among patients, particularly in relation to unhealthy opioid use (UOU)	COVID-19 and Unhealthy Opioid Use (UOU)	Not specified	machine learning techniques and mixed-effects ordinal logistic regression to assess the severity of COVID-19 outcomes in patients with UOU	Standard	No
712	34149017	Psychosocial Determinants of Readmission after Surgery	prospective cohort study	No	N/A	unplanned readmission to any medical facility within 30 days of hospital discharge	general, vascular, or thoracic surgery	Not specified	unadjusted and adjusted incidence rate ratios (IRRs) calculated using Poisson regression	Standard	Yes
713	36205670	More Accessible Covid-19 Treatment through Monoclonal Antibody Infusion in the Emergency Department	retrospective review	No	N/A	monoclonal antibody (MAB) infusion for COVID-19 treatment in an outpatient setting, specifically in the emergency department (ED)	COVID-19	Not specified	chi-squared tests for categorical variables and t-tests for continuous variables to compare two groups of patients receiving MAB infusion	Standard	No
714	37157005	Social Determinants of Health and Cdk4/6 Inhibitor Use and Outcomes among Patients with Metastatic Breast Cancer	retrospective study	Yes	systematic bias	the use and outcomes of CDK4/6 inhibitors (CDK4/6i) in patients with hormone receptor-positive, HER2-negative metastatic breast cancer (MBC)	Metastatic breast cancer (MBC)	Not specified	chi-square tests; multivariable logistic regression models	standard	No
715	34980656	Residential Segregation and Emergency Department Utilization among an Underserved Urban Emergency Department Sample in North Carolina	retrospective study	No	N/A	emergency department (ED) utilization	Not specified	Not specified	associations between community context and ED utilization, with interaction terms by race	standard	No
719	37087897	Exercise Facility Availability and Incidence of Type 2 Diabetes and Complications in Spain: A Population-Based Retrospective Cohort 2015-2018	retrospective cohort study	No	N/A	the incidence of type 2 diabetes	type 2 diabetes	Not specified	risk ratios (Poisson regression models). Interactions were explored with SES tertiles and by sex.	standard	No
722	28476461	Adding Social Determinant Data Changes Children's Hospitals' Readmissions Performance	retrospective cohort study	No	N/A	pediatric readmissions	all-condition readmissions in a pediatric hospital setting	Not specified	the change in hospitals' readmission decile-rank and the assessment of model fit and accuracy	standard	No
728	35211958	Risk from Delayed or Missed Care and Non-Covid-19 Outcomes for Older Patients with Chronic Conditions During the Pandemic	retrospective cohort study	No	N/A	COVID-19 pandemic	Chronic illnesses in older patients	Not specified	mortality, inpatient events, Medicare payments, and primary care and specialty care visits (both in-person and telehealth)	Standard	No

730	32927325	Insurance Type and Marital Status Impact Hospital Length of Stay after Pancreatoduodenectomy	Retrospective review of a prospective database	No	N/A	hospital length of stay (LOS) after pancreatoduodenectomy (PD)	pancreatoduodenectomy	Not specified	hospital LOS, insurance types, marital status, and various postoperative outcomes	Standard	No
731	33851193	Social and Clinical Determinants of Covid-19 Outcomes: Modeling Real-World Data from a Pandemic Epicenter	Retrospective cohort study	No	N/A	COVID-19 pandemic	COVID-19	Not specified	hospitalization, severe disease, and death due to COVID-19	standard	Yes
734	34593712	Assessing and Augmenting Predictive Models for Hospital Readmissions with Novel Variables in an Urban Safety-Net Population	Retrospective cohort study	No	N/A	Hospital readmissions	Not specified	Not specified	C-statistics and odds ratios for factors associated with readmissions	standard	yes
739	34159548	Neighborhood Effects on Missed Appointments in a Large Urban Academic Multispecialty Practice	Retrospective cross-sectional study	No	N/A	Missed appointments	Not specified	N/A	IV: demographic, patients' census tracts of residence, DV: Missed appointments	Standard (Goverment, city defined index/score like crime) and custom (survey questions to measure some SDoH, like perceived safety)	yes
744	28477050	Revealing the Prevalence and Consequences of Food Insecurity in Children with Epilepsy	Retrospective Observational Study	No	N/A	Acute healthcare utilization (ED visit or hospitalization)	Epilepsy	N/A	Association between FI and healthcare utilization, health-related quality-of-life (HR-QOL), and medication side effect control	Standard (ICD-10)	No
748	36720999	Influence of Social Deprivation Index on in-Hospital Outcomes of Covid-19	Retrospective cross-sectional study	No	N/A	Admission to ED or hospital	COVID-19	N/A	IV: Social Depriviation (including income, education, employment, housing, household characteristics and transportation); DV: in-hospital intubation and in-hospital mortality	Standard (Social Depriviation Index)	Yes
750	34038164	Prior Frequent Emergency Department Use as a Predictor of Emergency Department Visits after a New Cancer Diagnosis	Retrospective cohort study	No	N/A	Emergency department visits after a new cancer diagnosis	Cancer	N/A	Association between frequent ED use and ED visits after a new cancer diagnosis	Standard	Yes
766	34668658	Community Socioeconomic Deprivation Predicts Nonalcoholic Steatohepatitis	Retrospective observational study	No	N/A	Nonalcoholic Steatohepatitis	Nonalcoholic Steatohepatitis	N/A	Association between socioeconomic deprivation and nonalcoholic fatty liver disease (NAFLD)/nonalcoholic steatohepatitis (NASH)	Standard (ACS)	Yes
768	34360326	Psychosocial Determinants of Hiv Stigma among Men Who Have Sex with Men in San Francisco, California	Cross-sectional study	No	N/A	Not specified	HIV	N/A	IV: demographic, injection history, housing, mental health status, viral load; DV: experienced HIV stigma	Standard(IV) and custom(stigma)	No
777	35270315	Let It Beat: How Lifestyle and Psychosocial Factors Affect the Risk of Sudden Cardiac Death-a 10-Year Follow-up Study	Retro-prospective single-centre study	No	N/A	Implantable Cardioverter Defibrillator (ICD) intervention	Sudden cardiac death	Implantable Cardioverter Defibrillator (ICD)	IV: lifestyle and psychosocial factors; DV: sudden cardiac death risk	Standard (i.e., health information) and Custom (i.e., psychosocial factors = stress in personal life, stress in working life, and financial stress)	Yes
652	36847187	Utility of a Ml Analytics on Real Time Risk Stratification and Re-Intervention Risk Prediction on Av Access Outcomes and Cost	Single center analysis	No	N/A	Re-intervention risk prediction on AV access outcomes and cost	AVF and AV graft	N/A	Real time risk stratification and re-intervention risk prediction on AV access outcomes and cost	Custom	Yes

706	30198104	Development and Future Deployment of a 5 Years Allograft Survival Model for Kidney Transplantation	Retrospective cohort study	No	N/A	Allograft survival after kidney transplantation	Kidney transplantation	Yes	Five-year allograft survival	Custom	Yes
609	36214725	Unmet Needs for Food, Medicine, and Mental Health Services among Vulnerable Older Adults During the Covid-19 Pandemic	Observational cross-sectional study	No	N/A	Unmet needs for food, medicine, and mental health services	Not specified	Not specified	Regression analysis examining relationship between unmet needs for food, medicine and mental health services, and sociodemographic factors	Standard	No
666	33516898	Pediatric Primary Care-Based Social Needs Services and Health Care Utilization	Eetrospective cohort study	No	N/A	ambulatory, emergency, and inpatient services	Not specified	Not specified	adjusted incidence rate ratios	standard	No
683	35342984	Achieving Diabetes Treatment Targets in People with Registered Mental Illness Is Similar or Improved Compared with Those Without: Analyses of Linked Observational Datasets	Cross-sectional study	No	N/A	type 2 diabetes mellitus treatment targets	Type 2 diabetes mellitus	Not specified	multivariate logistic regression, odds ratio with confident intervals	standard	No
685	36276335	Using Phenome-Wide Association Studies and the Sf-12 Quality of Life Metric to Identify Profound Consequences of Adverse Childhood Experiences on Adult Mental and Physical Health in a Northern Nevadan Population	Cross-sectional study	No	N/A	Mental and physical health events of adverse childhood experiences		Not specified	Phenome-wide association studies, SF-12 quality of life metric.	standard	
720	36569128	Inequalities in Glycemic and Multifactorial Cardiovascular Control of Type 2 Diabetes: The Heart Healthy Hoods Study	Cross-sectional study	No	N/A	Glycemic and multifactorial cardiovascular control in Type 2 Diabetes	type 2 diabetes	Not specified	Factors associated with glycemic control using mutilevel logistic regression	standard	No
737	35360124	Contributions of Covid-19 Pandemic-Related Stressors to Racial and Ethnic Disparities in Mental Health During Pregnancy	Cross-sectional study	No	N/A	Mental health during pregnancy in Covid-19 pandemic	prenatal depression and anxiety	Not specified	Adjusted prevalence ratio for prenatal depression and anxiety among different racial and ethnic groups	standard	No
762	36931379	Heat-Related First Cardiovascular Event Incidence in the City of Madrid (Spain): Vulnerability Assessment by Demographic, Socioeconomic, and Health Indicators	Restrospective Observational study	No	N/A	acute cardiovascular events	cardiovascular diseases	Not specified	odds ratio association between heat and the incidence of first acute vardiovascular events	standard	No

EndNote_I	PubMed_I	Title	Types of Intervention	Intervention level	Family/Social suppurt?	what type of health outcomes	Intervention effectiveness	Generalizability
332	26391941	A Road Map to Address the Social Determinants of Health through Community Collaboration	Social programs - Community health initiatives, educational programs	Community level - Community health outreach, local health education campaigns	Yes - Family-centered approaches, family counseling, family	Health outcomes - Improvement in health metrics, reduction in disease incidence	Demographic diversity - Effectiveness across age groups, ethnicities, socioeconomic statuses	
379	30649280	Screening for Poverty and Intervening in a Primary Care Setting: An Acceptability and Feasibility Study	Social programs	Hospital level	No	Provider perspectives on acceptability and feasibility	Unclear	
445	36457714	Impact of a Mobile Integrated Healthcare and Community Paramedicine Program on Improving Medication Adherence in Patients with Heart Failure and Chronic Obstructive Pulmonary Disease after Hospital Discharge: A Pilot Study	Resource allocation - Allocation of medical staff and equipment	Hospital level - Implemented after hospital discharge	No	Health outcomes - Medication adherence	Unclear - Small sample size from one hospital	
497	36068611	Needs Assessment and Planning for a Clinic-Community-Based Implementation Program for Hypertension Control among Blacks in New York City: A Protocol Paper	Social programs - Community health initiatives	Community level - Community health outreach	No	None - This is a protocol paper	Not reported - Protocol paper	
525	20820029	Medical-Legal Partnerships: Transforming Primary Care by Addressing the Legal Needs of Vulnerable Populations	Social programs - Medical-legal partnerships	Community level - Integrated into primary care	No	Not reported - Descriptive article	Not reported - Descriptive article	
540	31749028	Impact of a Low-Intensity Resource Referral Intervention on Patients' Knowledge, Beliefs, and Use of Community Resources: Results from the Communityrx Trial	Social programs - Resource referral intervention	Hospital level - Implemented in primary care clinic and emergency department	No	Knowledge and beliefs about community resources, resource self-efficacy, resource utilization	Unclear - Single health system in one geographic area	
543	33468104	The Mitigating Toxic Stress Study Design: Approaches to Developmental Evaluation of Pediatric Health Care Innovations Addressing Social Determinants of Health and Toxic Stress	Social programs - Pediatric clinic innovations to address social determinants of health	Hospital level - Implemented in pediatric clinics	Yes - Family-centered approaches	Not reported - Study protocol	Unclear - Study protocol across multiple clinics and communities	
552	32527322	Enhanced Care Planning and Clinical-Community Linkages Versus Usual Care to Address Basic Needs of Patients with Multiple Chronic Conditions: A Clinician-Level Randomized Controlled Trial	Social programs - Enhanced care planning and community linkages	Community level - Implemented in primary care clinics	No	Health outcomes - Control of chronic conditions, quality of life	Unclear - Single health system in one geographic area	
557	32449443	Impact of an on-Site Versus Remote Patient Navigator on Pediatricians' Referrals and Families' Receipt of Resources for Unmet Social Needs	Resource allocation - Patient navigator	Hospital level - Implemented in pediatric clinics	No	Referrals to patient navigator, contact with families, enrollment in resources	Unclear - Single patient navigator across few clinics	
584	32392126	Evaluation of the Learning to Integrate Neighborhoods and Clinical Care Project: Findings from Implementing a New Lay Role into Primary Care Teams to Address Social Determinants of Health	Social program - Implementation of a community health worker role	Community/clinic level - Intervention implemented in primary care clinics	No specific family or social support component	Patient satisfaction, health outcomes (focus groups indicated improved health, but no changes detected in surveys or administrative data)	Unclear generalizability due to limited sample size and lack of randomization; provides lessons learned for implementing similar roles	

603	30562141	Mechanisms Associated with Clinical Improvement in Interventions That Address Health-Related Social Needs: A Mixed-Methods Analysis	Social program - Linking patients to community resources	Community/clinic level - Embedded in primary care clinics	No specific family or social support component	Health outcomes (blood pressure, LDL, A1c), patient satisfaction, resource utilization	Unclear generalizability due to single health system; provides qualitative insights into mechanisms
608	36754720	Reducing Barriers to Covid-19 Vaccine Uptake through a Culturally Sensitive Pharmacy-Led Patient Medication Education Group in a Behavioral Health Population	Social program - Culturally sensitive pharmacy-led education program	Hospital level - Behavioral health facility	No specific family or social support component	Health outcomes (vaccine uptake rates), patient satisfaction	Unclear generalizability due to single site; provides insights into addressing vaccine hesitancy
632	33779065	Use of an Integrated Care Management Program to Uncover and Address Social Determinants of Health for Individuals with Lupus	Social program - Integrated care management program	Hospital level - Academic medical center	No specific family or social support component	Patient satisfaction, access and equity (uncovering and addressing social needs)	Unclear generalizability due to single academic medical center
670	35759702	Nonmedical Interventions for Type 2 Diabetes: Evidence, Actionable Strategies, and Policy Opportunities	Social programs, policy changes	Community, city/regional, hospital	No specific family or social support	Health outcomes (HbA1c, blood pressure, cholesterol), access and equity	Unclear generalizability due to limited evidence base; provides framework for policy opportunities
688	36999452	2022 Ashp Survey of Health-System Specialty Pharmacy Practice: Clinical Services	Resource allocation - Health system specialty pharmacy services	Hospital level - Within health systems	No specific family or social support	Patient satisfaction, health outcomes, economic (cost savings)	Unclear generalizability due to convenience sample; provides overview of services offered by health system specialty pharmacies
691	32548774	Demonstrated Health Care Cost Savings for Women: Findings from a Community Health Worker Intervention Designed to Address Depression and Unmet Social Needs	Social program - Community health worker intervention	Hospital/clinic level - Urban women's health clinics	No specific family/social support component	Economic (healthcare costs), access and equity (unmet social needs)	Single health system - unclear generalizability; provides evidence for cost savings of CHW intervention
718	32305457	Translating Research into Practice: Protocol for a Community-Engaged, Stepped Wedge Randomized Trial to Reduce Disparities in Breast Cancer Treatment through a Regional Patient Navigation Collaborative	Social program - Patient navigation services	Hospital level - Academic medical centers	No specific family or social support	Health outcomes (treatment timeliness, guideline concordance), implementation outcomes (acceptability, adoption, fidelity, sustainability, cost)	Unclear generalizability as the protocol is for a single region; provides model for addressing disparities through patient navigation
721	31460769	Group Medical Visits 2.0: The Open Source Wellness Behavioral Pharmacy Model	Social program - Group education and coaching sessions	Community level - Federally qualified health center	Yes - Peer support and social connections emphasized	Health outcomes (blood pressure, BMI, depression), access and equity (targeting underserved populations)	Single health center - unclear generalizability; provides model for behavioral pharmacy
760	36054022	Targeting Technology in Underserved Adults with Type 1 Diabetes: Effect of Diabetes Practice Transformations on Improving Equity in Cgm Prescribing Behaviors	Resource allocation - Diabetes practice transformations	Hospital level - Endocrinology clinics	No specific family or social support	Health outcomes (CGM prescription rates), access and equity (focus on underserved populations)	Single health system - unclear generalizability; provides model for practice transformations
708	37007375	Effects of California's New Patient Homelessness Screening and Discharge Care Law in an Emergency Department	Policy Changes	Hospital Level	No specific mention	Health Outcomes (ED visit rates, demographic shifts), Access and Equity (identification of homeless	Demographic Diversity (age, sex, race), Geographic Transferability (specific to suburban California ED)
534	36704207	Evaluating a Social Risk Screening and Referral Program in an Urban Safety-Net Hospital Emergency Department	Social program - Social Risk Screening and Referral Program	Hospital Level	No	Patient's perspectives on acceptability and feasibility	Not reported - Feasibility paper

Type of intervention (e.g., policy changes, social programs, resource allocation).
Level at which the intervention is targeted (e.g., hospital, community, city).
Incorporation of family or social support in the intervention.
Metrics used to determine the effectiveness of the intervention.
Generalizability of the intervention outcomes.
